# Transcriptomic Insights into GABA Accumulation in Tomato via CRISPR/Cas9-Based Editing of *SlGAD2* and *SlGAD3*

**DOI:** 10.3390/genes16070744

**Published:** 2025-06-26

**Authors:** Jin-Young Kim, Yu-Jin Jung, Dong Hyun Kim, Kwon-Kyoo Kang

**Affiliations:** 1 Division of Horticultural Biotechnology, Hankyong National University, Anseong 17579, Republic of Korea; zino@hknu.ac.kr (J.-Y.K.); yuyu1216@hknu.ac.kr (Y.-J.J.); skullmask@naver.com (D.H.K.); 2 Institute of Genetic Engineering, Hankyong National University, Anseong 17579, Republic of Korea

**Keywords:** CRISPR/Cas9, GABA, glutamate decarboxylase (GAD), tomato fruit, metabolic engineering, transcriptome

## Abstract

Background: *γ*-Aminobutyric acid (GABA) is a non-proteinogenic amino acid with key roles in plant metabolism, stress responses, and fruit nutritional quality. In tomato (*Solanum lycopersicum*), GABA levels are dynamically regulated during fruit development but decline in the late ripening stages. Methods: To enhance GABA accumulation, we used CRISPR/Cas9 to edit the calmodulin-binding domain (CaMBD) of *SlGAD2* and *SlGAD3*, which encode glutamate decarboxylases (GADs). The resulting truncated enzymes were expected to be constitutively active. We quantified GABA content in leaves and fruits and performed transcriptomic analysis on edited lines at the BR+7 fruit stage. Results: CaMBD truncation significantly increased GABA levels in both leaves and fruits. In *gad2* sg1 lines, GABA levels increased by 3.5-fold in leaves and 3.2-fold in BR+10 fruits; in *gad3* sg3 lines, increases of 2.8- and 2.5-fold were observed, respectively. RNA-seq analysis identified 1383 DEGs in *gad2* #1−5 and 808 DEGs in *gad3* #3−8, with 434 DEGs shared across both lines. These shared DEGs showed upregulation of *GAD*, *GABA-T*, and *SSADH*, and downregulation of stress-responsive transcription factors including *WRKY46*, *ERF*, and *NAC*. Notably, total free amino acid content and fruit morphology remained unchanged despite elevated GABA. Conclusions: CRISPR/Cas9-mediated editing of the CaMBD in *SlGAD* genes selectively enhances GABA biosynthesis in tomato without adverse effects on development or fruit quality. These lines offer a useful platform for GABA-centered metabolic engineering and provide insights into GABA’s role in transcriptional regulation during ripening.

## 1. Introduction

Gamma-aminobutyric acid (GABA) is a non-protein amino acid that functions as the principal inhibitory neurotransmitter in the mammalian central nervous system, where it modulates synaptic transmission and contributes to neural homeostasis [1]. Beyond its physiological role in animals, GABA has emerged as a health-promoting compound with documented benefits in reducing blood pressure, alleviating stress, and enhancing immune responses upon dietary intake [2,3]. As a result, GABA-enriched functional foods—including dietary supplements and fermented beverages—have gained popularity in the nutraceutical industry [4,5]. In plants, GABA is primarily synthesized through the GABA shunt, a conserved metabolic route that bypasses the tricarboxylic acid (TCA) cycle. This pathway involves three core enzymes: glutamate decarboxylase (GAD), GABA transaminase (GABA-T), and succinic semialdehyde dehydrogenase (SSADH) [6,7]. GAD catalyzes the decarboxylation of glutamate into GABA in the cytosol, effectively channeling carbon into the TCA cycle via succinate production [8]. The GABA shunt contributes to diverse plant physiological functions, including pH regulation, C/N homeostasis, oxidative stress response, energy balance, and adaptation to biotic and abiotic stresses [7,9]. GABA accumulation is known to be highly inducible under environmental stimuli such as heat, hypoxia, mechanical wounding, and hormonal signals [10]. Plant GAD enzymes possess a conserved C-terminal calmodulin-binding domain (CaMBD), which negatively regulates enzymatic activity in the absence of Ca^2+^/calmodulin interaction. Truncation or mutation of this CaMBD has been shown to relieve this suppression, thereby enhancing GABA biosynthesis in transgenic plants [11,12]. In tomato, three glutamate decarboxylase (GAD) isoforms—*SlGAD1*, *SlGAD2*, and *SlGAD3*—have been identified. Among these, *SlGAD2* and *SlGAD3* are more highly expressed in fruit tissues, suggesting that they play specific roles in fruit-associated GABA biosynthesis [13]. Previous studies have demonstrated that CRISPR/Cas9-mediated editing of *SlGAD2* and *SlGAD3*—introducing premature stop codons upstream of the CaMBD—effectively elevates GABA content in tomato fruit by generating truncated GAD proteins [14]. However, the broader consequences of such editing on fruit metabolism and gene expression remain poorly understood. In particular, the distinct contributions of *SlGAD2* and *SlGAD3* to amino acid metabolism, their regulatory impact on global transcriptome profiles, and the integration of metabolomic and transcriptomic responses have not been comprehensively addressed.

In this study, we engineered CRISPR/Cas9 constructs targeting the C-terminal region of *SlGAD2* and *SlGAD3* to disrupt CaMBD domains and enhance GABA accumulation. Using transgenic tomato lines, we investigated the effects of GAD editing on GABA levels, free amino acid composition, and transcriptomic alterations in fruit tissues. Our results provide mechanistic insights into GABA regulation and highlight the utility of targeted gene editing for developing GABA-enriched functional tomato cultivars with improved nutritional properties.

## 2. Materials and Methods

### 2.1. Development of CRISPR/Cas9-Edited Tomato Lines and Fruit Tissue Sampling

CRISPR/Cas9-mediated genome editing was performed to generate tomato lines targeting *SlGAD2* (Solyc11g011920) and *SlGAD3* (Solyc01g005000) in the inbred cultivar ‘K19’. sgRNAs were designed using CRISPR RGEN Tools Cas-Designer [15], synthesized by Bioneer, and cloned into the *Aar*I-digested pKAtC binary vector. The constructs were introduced into *Agrobacterium tumefaciens* EHA105 and transformed into cotyledon explants. Transgenic shoots were selected on an MS medium with 100 mg/L kanamycin, rooted, and transferred to soil. T_0_ plants were screened by PCR and self-pollinated to obtain homozygous T_1_ lines. All plants were grown under controlled greenhouse conditions. Fruits were harvested at the mature green (MG), breaker (BR), and 7 and 10 days after breaker (BR+7, BR+10) stages. Pericarp and leaf samples were collected, frozen in liquid nitrogen, and stored at −80 °C for further analyses. Primer sequences used for genotyping are listed in Supplementary Table S2.

### 2.2. Analysis of GABA and Total Amino Acids

The contents of γ-aminobutyric acid (GABA) and total free amino acids were measured according to the method of Kim et al. [16], with minor modifications. Lyophilized tomato powder (250 mg) was extracted three times with 10 mL of 70% ethanol using ultrasonic treatment (10 min each). The combined extracts were centrifuged at 5700× *g* for 10 min, and the supernatant was filtered through a 0.22 μm membrane. GABA and total amino acids were quantified using a Biochrom 30+ amino acid analyzer (Biochrom Ltd., Cambridge, UK), with ninhydrin-based post-column derivatization and absorbance detection at 570 nm.

### 2.3. RNA Extraction and Transcriptome Sequencing

Total RNA was extracted from pericarp tissues of wild-type and CRISPR-edited tomato fruits at the breaker stage using TRIzol reagent (Invitrogen, Carlsbad, CA, USA); each genotype was represented by three biological replicates. RNA integrity was first checked on a 1.2% agarose gel and then confirmed on an Agilent 2100 Bioanalyzer (RIN > 7.0 for all samples). RNA-seq libraries were prepared with the NEBNext^®^ Ultra™ RNA Library Prep Kit and sequenced on an Illumina HiSeq PE150 platform (Novogene, Tianjin, China), yielding ≥ 4 Gb of 150 bp paired-end data per sample. Raw reads were assessed with FastQC (v0.11.9) and adapter/low-quality bases (Phred < 20) were removed using Trimmomatic (v0.39; ILLUMINACLIP:2:30:10, SLIDINGWINDOW:4:20, MINLEN:36). Reads < 90 bp after trimming were discarded. High-quality reads were aligned to the tomato reference genome (SL2.40) using TopHat (v1.4.6; --read-mismatches 2, --max-multihits 1), and only primary alignments were retained with SAMtools (v1.10). Gene-level counts were generated with FeatureCounts (v2.0.14; minOverlap 30). For differential-expression analysis, genes with < 10 total counts across all samples were removed to reduce noise. Raw counts were imported into DESeq2 (v1.26.0) and transformed using the variance-stabilizing transformation (vst) for exploratory analyses (e.g., PCA). DEGs were identified with the Wald test (design formula = ~Genotype); genes with |log_2_FC| ≥ 1 (fold change ≥ 2) and an adjusted *p*-value (Benjamini–Hochberg) < 0.05 were deemed significant. Expression values were normalized as FPKM for presentation, while DEG statistics were derived from raw counts. Functional enrichment of DEGs was carried out with ClusterProfiler (v3.10.1) for Gene Ontology categories (adjusted *p* < 0.05). Heatmaps and volcano plots were generated with pheatmap and EnhancedVolcano, respectively, providing an overview of transcriptomic changes between wild-type and edited lines.

### 2.4. Quantitative Reverse Transcription PCR (qRT-PCR) Analysis

Total RNA was isolated from tomato pericarp tissue using the RNeasy Plant Mini Kit (Qiagen, Hilden, Germany) according to the manufacturer’s instructions. First-strand cDNA was synthesized from 1 μg of total RNA using a reverse transcription kit with random hexamer primers (Takara Bio Inc., Seoul, Republic of Korea). Quantitative real-time PCR was performed using gene-specific primers and SYBR Green Real-time PCR Master Mix (Toyobo, Osaka, Japan) on a real-time PCR detection system. Relative expression levels were calculated using the 2^−ΔΔCt^ method, with *SlActin* as the internal control. Each biological sample was analyzed in three biological replicates, and each reaction was performed in technical triplicates. Primer sequences used in this study are provided in Supplementary Table S2. The procedure followed the method described by Kim et al. [17].

### 2.5. Statistical Analysis

All statistical analyses were conducted using R software (version 4.3.1). Experiments were performed with three independent biological replicates unless otherwise stated. To evaluate differences in phenotypic traits and gene expression levels, one-way analysis of variance (ANOVA) was applied, followed by Tukey’s Honestly Significant Difference (HSD) test for post hoc comparisons. Statistical significance was considered at *p* < 0.05 (*), *p* < 0.01 (**), and *p* < 0.001 (***). Data are presented as mean ± standard deviation (SD).

## 3. Results

### 3.1. Generation and Molecular Characterization of SlGAD2 and SlGAD3 CRISPR/Cas9-Edited Lines

**Figure 1:**
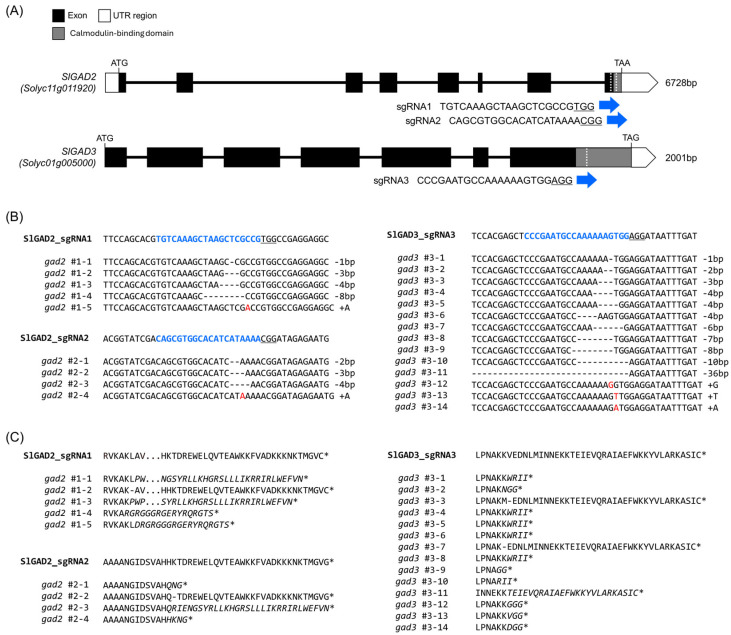
CRISPR/Cas9-mediated editing of *SlGAD2* and *SlGAD3* in tomato. (**A**) Schematic representation of the gene structures of *SlGAD2* and *SlGAD3*, showing the positions of the sgRNA target sites (blue arrows) near the 3′ end of the coding sequences. Exons are indicated by boxes, with dark gray representing coding sequences (CDS) and white indicating untranslated regions (UTRs). The target sequences for each sgRNA are shown in blue. (**B**) Deep sequencing analysis of the CRISPR target regions in transgenic plants. Blue text indicates sgRNA sequences; hyphen (-) represent deleted nucleotides; red letters indicate inserted bases. (**C**) Translated amino acid sequences of the corresponding mutant alleles. Asterisks (*) denote premature stop codons, and italicized residues indicate altered amino acid sequences resulting from frameshift mutations.

The C-terminal calmodulin-binding domains (CaMBDs) of *SlGAD2* and *SlGAD3* were selectively disrupted by targeting their 3′ coding regions with guide RNAs (Figure 1A, Table S1). The selected sgRNAs induced double-strand breaks upstream of the CaMBD-encoding sequences, thereby promoting frameshift mutations and premature stop codons through nonhomologous end joining (NHEJ)-mediated repair. These constructs were introduced into the tomato cultivar ‘K19’ via *A. tumefaciens*-mediated transformation, and primary transgenic (T_0_) lines were selected based on kanamycin resistance (Figure S1). Deep sequencing of the target sites revealed diverse mutation patterns across the edited *SlGAD2* and *SlGAD3* alleles (Figure 1B, Table S3). In *SlGAD2*, editing by sgRNA1 resulted in −8 bp deletions and single adenine (A) insertions, both of which caused frameshifts leading to premature stop codons. Likewise, sgRNA2-induced edits included −2 bp deletions and A insertions, also generating early termination signals. In *SlGAD3*, most edited alleles contained frameshift mutations that introduced premature stop codons upstream of the CaMBD, except for rare in-frame deletions (multiples of three nucleotides) that preserved the open reading frame. Translation of the mutated coding sequences confirmed that most edited alleles encoded truncated GAD proteins lacking the CaMBD (Figure 1C). These truncations are predicted to relieve the enzyme from calmodulin-dependent negative regulation, thereby enhancing GAD activity and promoting GABA biosynthesis. All T_0_ transformants were self-pollinated, and segregating T_1_ progenies were genotyped to identify individuals homozygous for the respective mutant alleles. The diverse editing patterns—including various insertions and deletions—were successfully fixed in homozygous lines, which were subsequently used for all downstream physiological, biochemical, and transcriptomic analyses.

### 3.2. Phenotypic Characterization of Homozygous SlGAD2 and SlGAD3 Edited Lines

**Figure 2:**
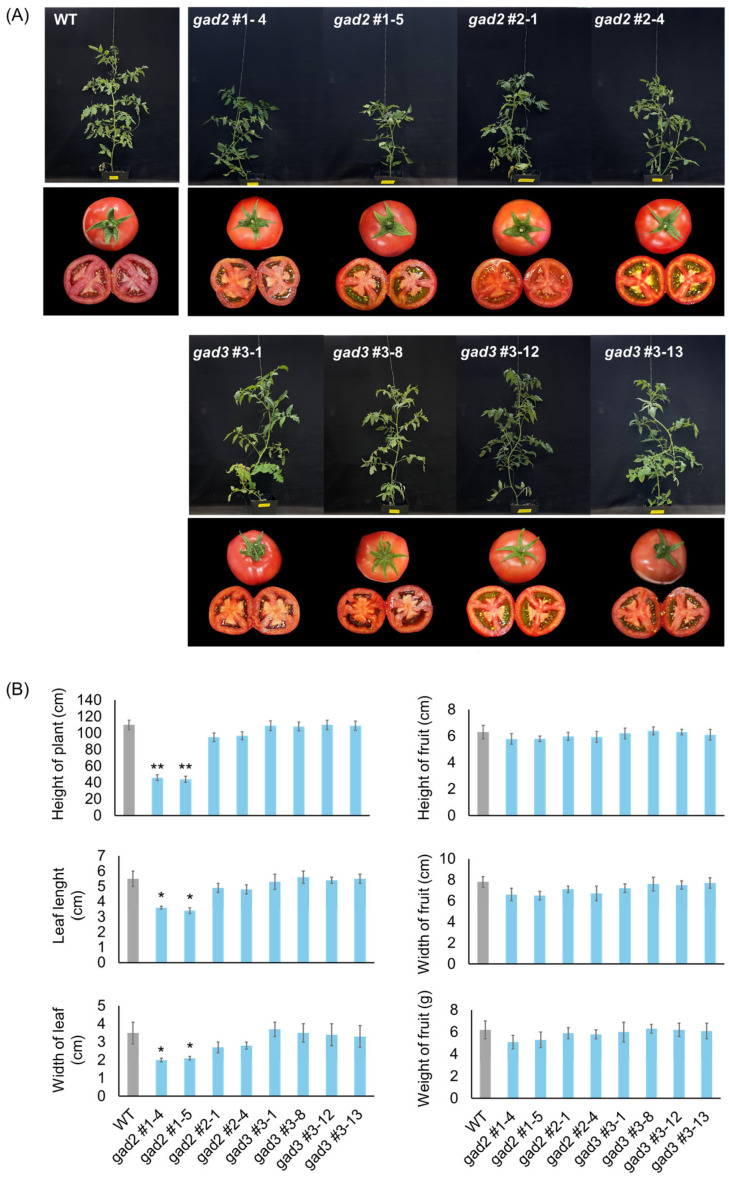
Phenotypic analysis of CRISPR/Cas9-edited *SlGAD2* and *SlGAD3* tomato lines. (**A**) Representative images of WT and *SlGAD*-edited lines at the mature vegetative stage (upper panels) and of harvested fruits (lower panels). Scale bars = 2.5 cm. (**B**) Quantitative analysis of morphological traits, including plant height, leaf width and length, fruit width, length, and fresh weight. Bars represent means ± SD (n = 6). Asterisks indicate significant differences from the wild type (* *p* < 0.05, ** *p* < 0.01).

Homozygous *SlGAD2* and *SlGAD3* mutant lines (T_1_) were self-fertilized, and individual seeds were advanced to the T_2_ generation. Transgene-free null segregants were identified via kanamycin sensitivity assays and PCR-based genotyping (Figure S2), allowing phenotypic analyses to be performed in stable, edited backgrounds. Morphological assessment of these null lines revealed distinct alterations in vegetative traits, particularly in the *gad2* sg1 line. As shown in Figure 2A, *gad2* sg1 plants exhibited a significant reduction in plant height and leaf size compared to the wild type. A moderate decrease was also noted in *gad2* sg2, although the differences were not statistically significant. In contrast, *gad3* mutants showed no visible phenotypic deviations from WT controls under standard greenhouse conditions. Quantitative data on plant height, leaf width and length, and fruit morphology (including width, length, and weight) are summarized in Figure 2B. In *gad2* sg1, both plant height (*p* < 0.01) and leaf dimensions (*p* < 0.05) were significantly reduced, whereas fruit-related traits remained unchanged across all mutant lines. These results indicate that *SlGAD2*, especially the sg1-targeted allele, contributes to the regulation of vegetative development without significantly affecting fruit morphology.

### 3.3. GABA and Free Amino Acid Profiling in Edited Tomato Fruit

**Figure 3:**
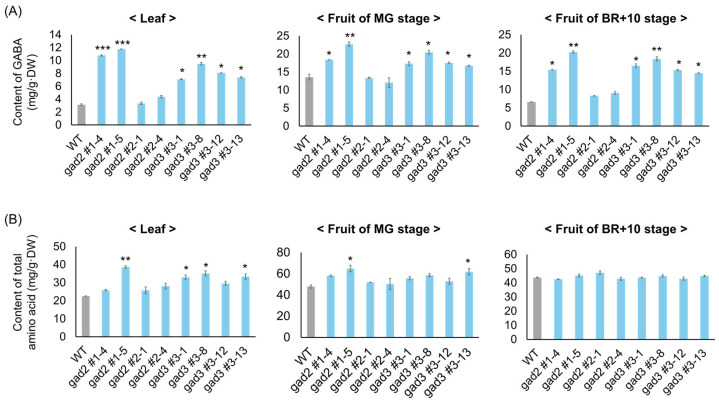
Quantification of GABA and total free amino acids in *SlGAD* edited lines. (**A**) GABA content in leaf and fruit tissues from wild-type and *SlGAD*-edited lines. (**B**) Total free amino acid levels in the same samples. Fruits were harvested at MG and 10 days after breaker stage. Bars represent means ± SD (n = 3). Asterisks indicate significant differences from the wild type (* *p* < 0.05, ** *p* < 0.01, *** *p* < 0.001).

The impact of *SlGAD2* and *SlGAD3* editing on GABA metabolism was examined by quantifying γ-aminobutyric acid (GABA) and total free amino acid (FAA) contents in three tissue types: leaves, mature green (MG) fruits, and fruits at 10 days after breaker (BR+10) (Figure 3). GABA levels were significantly elevated in all edited lines relative to the wild type (Figure 3A). In leaves, *gad2* sg1 lines #1–4 and #1–5 showed the greatest increases, with 3.2-fold and 3.5-fold elevations, respectively. *gad3* sg3 lines #3–1, #3–8, #3–12, and #3–13 also exhibited enhanced GABA accumulation, ranging from 2.1- to 2.8-fold (2.1-fold for #3–1, 2.3-fold for #3–8, 2.6-fold for #3–12, and 2.8-fold for #3–13). In MG fruits, GABA concentrations increased in all lines except *gad2* sg2, with the highest levels observed in *gad2* sg1 #1–5 (3.1-fold) and *gad3* sg3 #3–8 (2.7-fold). Other edited lines displayed 1.8- to 2.5-fold increases. Although GABA typically declines during fruit ripening, elevated levels were maintained at BR+10 in several edited lines: *gad2* #1–4 and #1–5 remained 2.9-fold and 3.2-fold higher than the wild type, respectively, and *gad3* #3–8 retained a 2.5-fold increase. This sustained accumulation suggests that truncation of the CaM-binding domain mitigates GABA catabolism during ripening. In contrast, FAA levels exhibited more moderate and tissue-dependent changes (Figure 3B). In leaves, *gad2* #1–5 showed a 1.6-fold increase, while *gad3* lines #3–1, #3–12, and #3–13 exhibited 1.3- to 1.5-fold increases. In MG fruits, FAA content was slightly elevated in *gad2* #1–5 (1.4-fold) and *gad3* #3–13 (1.3-fold). By the BR+10 stage, FAA levels in all edited lines were largely unchanged relative to the wild type, with fold changes ranging from 1.0 to 1.1, indicating minimal impact on global amino acid pools during late fruit development.

### 3.4. Transcriptomic Profiling of SlGAD2 and SlGAD3 Mutants Highlights GABA-Associated Pathways

**Figure 4:**
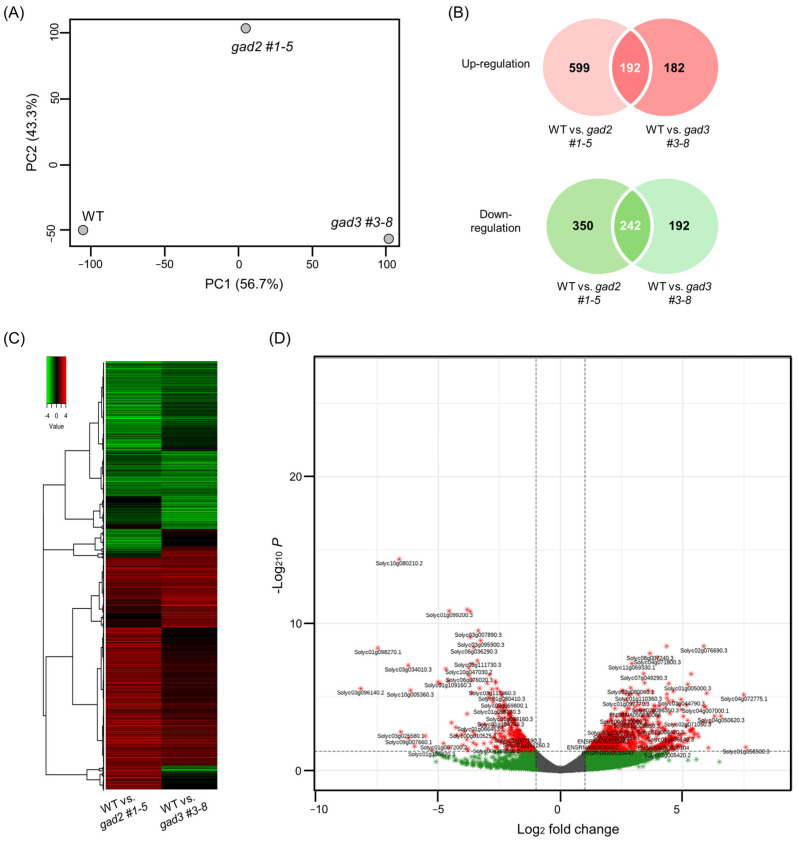
Shared transcriptomic response in *SlGAD2* and *SlGAD3* edited tomato fruits at the BR+7 stage. (**A**) Principal component analysis (PCA) of RNA-seq data derived from pericarp tissues of red ripe fruits at the BR+7 stage. Samples from *gad2* #1–5, *gad3* #3–8, and WT cluster distinctly, indicating genotype-dependent transcriptional profiles. (**B**) Venn diagrams showing the number of differentially expressed genes (DEGs) that are commonly upregulated (top) or downregulated (bottom) in both *gad2* #1–5 and *gad3* #3–8 lines compared to WT. DEGs were identified using DESeq2 with thresholds of |log_2_FC| ≥ 1 and adjusted *p*-value < 0.05. (**C**) Heatmap of the 434 shared DEGs (192 upregulated, 242 downregulated), visualized via hierarchical clustering. Red indicates relatively upregulated genes, while green indicates downregulation relative to wild type. Samples cluster by genotype, reflecting consistent expression patterns across biological replicates. (**D**) Volcano plot depicting the distribution of the 434 shared DEGs by log_2_ fold change and statistical significance. Genes meeting the cutoff criteria (adjusted *p*-value < 0.05 and |log_2_FC| ≥ 1) are highlighted in red (upregulated) and green (downregulated). Selected DEGs with strong statistical significance and high expression changes are labeled by gene ID for reference.

Transcriptomic alterations associated with GABA accumulation were characterized by performing RNA-seq on red ripe tomato fruits collected at 7 days after BR+7 from *SlGAD2*- and *SlGAD3*-edited lines (*gad2* #1–5, *gad3* #3–8) alongside WT controls. The BR+7 stage was chosen to capture late-stage developmental gene activity, as global transcription declines markedly by BR+10, limiting the detection of biologically meaningful changes. Sequencing generated 10.2–11.5 million high-quality reads per sample, with >95.8% mapping efficiency across all libraries (Table S4). Differential expression analysis revealed a broader transcriptomic shift in *gad2* compared to *gad3*, with 1383 differentially expressed genes (DEGs; 791 upregulated and 592 downregulated) and 808 DEGs (374 upregulated and 434 downregulated), respectively. MA plots (Figure S3) and principal component analysis (Figure 4A) consistently indicated that *gad2* lines were more transcriptionally divergent from WT, suggesting that *SlGAD2* disruption exerts a greater systemic effect than *SlGAD3*. A set of 434 DEGs (192 upregulated and 242 downregulated) was found to be commonly regulated in both edited lines (Figure 4B). Hierarchical clustering of these genes clearly separated mutant from WT samples (Figure 4C), and volcano plot analysis (Figure 4D) highlighted DEGs with the strongest statistical and biological relevance. Among the most notable transcriptional changes were genes involved in the GABA shunt. Both *Solyc01g005000.3.1* (glutamate decarboxylase, GAD) and *Solyc03g078150.3.1* (amino acid transporter family protein) were significantly upregulated, indicating enhanced glutamate turnover and increased GABA flux. These transcriptional changes support the observed rise in GABA levels and suggest that *SlGAD* editing triggers a shift toward GABA biosynthesis as a core metabolic outcome. Genes related to stress responses also showed coordinated expression changes. Several transcription factors associated with abiotic stress signaling—including *NAC*, *WRKY*, and *ERF* family members—were broadly downregulated, while a drought-inducible zinc finger gene (*Solyc04g007470.3.1*) was consistently induced in both lines. This pattern suggests a potential reprogramming from general stress signaling toward a GABA-modulated adaptive response under altered metabolic conditions. Genes involved in membrane ion homeostasis were also differentially expressed, though changes were more modest. The inward potassium channel *KAT1* (*Solyc08g016500.3.1*) was upregulated, whereas several calcium- and potassium-associated transporters, including *PBP1* and *GORK*, were repressed. These alterations may reflect the downstream effects of elevated GABA on ion flux and osmotic regulation, in line with its known role as a signaling molecule involved in membrane stability during stress. Collectively, these results indicate that *SlGAD2* and *SlGAD3* editing elicits a convergent transcriptomic program marked by enhanced GABA biosynthesis and restructured stress-responsive gene networks. This coordinated reprogramming likely underlies the physiological and metabolic adaptations observed in GABA-enriched tomato fruits during late ripening. A summary of key DEGs is presented in Table 1, with the complete dataset available in Table S5.

### 3.5. qRT-PCR Validation of Differentially Expressed Genes Related to GABA Metabolism and Stress Signaling

**Figure 5:**
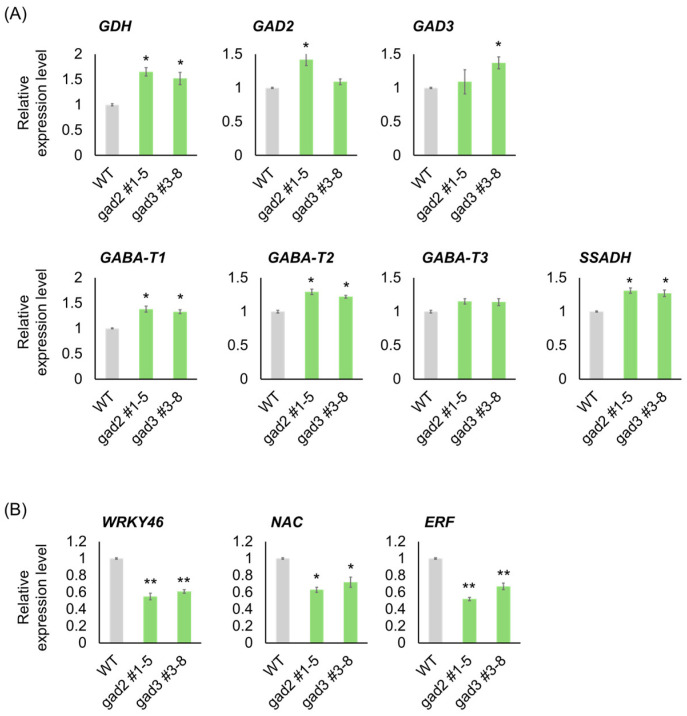
Validation of GABA Pathway and Stress-Responsive Genes by qRT-PCR. (**A**) Expression levels of GABA metabolism-related genes including (**B**) Expression of stress-responsive transcription factor genes *WRKY46*, *NAC*, and *ERF*. qRT-PCR analysis was performed using fruit samples harvested at the BR+7 stage. Bars represent means ± SD (n = 3). Asterisks indicate significant differences from the wild type (* *p* < 0.05, ** *p* < 0.01).

Quantitative RT-PCR analysis was performed to validate the expression patterns of genes involved in GABA metabolism and stress signaling, as identified by RNA-seq. As shown in Figure 5A, genes associated with the GABA shunt were consistently upregulated in both *gad2* #1–5 and *gad3* #3–8 mutants relative to the WT. *GAD2* and *GAD3* transcript levels were moderately elevated in their respective edited lines despite C-terminal truncation, possibly due to feedback activation under increased GABA concentrations. *GDH* expression exhibited a significant increase (~1.5-fold), consistent with enhanced glutamate turnover. *GABA-T1* and *GABA-T2* were clearly upregulated, whereas *GABA-T3* remained unchanged. *SSADH*, the terminal enzyme in the GABA shunt, was also significantly induced in both mutants, reinforcing the conclusion that GABA metabolic flow was upregulated. In contrast, stress-responsive transcription factors showed significant downregulation in the edited lines (Figure 5B). *WRKY46* and *ERF* were strongly repressed, with transcript levels reduced by approximately 40–50%, while *NAC* expression was moderately but significantly decreased. These findings support the interpretation that elevated GABA levels—driven by constitutive GAD activity—may suppress classical abiotic stress signaling pathways during late fruit ripening.

## 4. Discussion

CRISPR/Cas9-mediated editing of *SlGAD2* and *SlGAD3* successfully truncated their CaMBDs, resulting in frameshift alleles that encode catalytically active yet regulation-relieved GAD proteins. These results are consistent with prior studies demonstrating that CaMBD truncation abolishes calmodulin-mediated inhibition, thereby enhancing GAD activity and promoting GABA overproduction [18,19]. In the present study, the majority of edited alleles introduced premature stop codons upstream of the CaMBD (Figure 1B), and in silico translation confirmed the absence of this domain in most T_0_ and T_1_ lines (Figure 1C). The physiological effects of these edits were particularly pronounced in *SlGAD2*-edited lines. Specifically, *gad2* sg1 mutants exhibited significantly reduced plant height and smaller leaves (Figure 2A,B), while *gad3* mutants remained phenotypically comparable to wild type under standard greenhouse conditions. These observations suggest a broader developmental role for *SlGAD2*, possibly mediated through GABA-associated growth regulatory pathways [20]. Prior research has shown that GABA modulates developmental and stress-responsive processes by altering cytosolic pH, calcium signaling, and hormonal crosstalk [21,22], which may explain the growth phenotype uniquely observed in *SlGAD2*-edited plants. GABA levels were markedly elevated in both *SlGAD2* sg1 and *SlGAD3* sg3 lines across all sampled tissues and fruit developmental stages (Figure 3A). In leaves, GABA content increased up to 3.5-fold, while fruits at the MG and BR+10 stages maintained 2.5- to 3.2-fold increases. This sustained accumulation during ripening contrasts with the typical decline in wild-type fruits, suggesting that CaMBD truncation not only activates GAD enzymatic function but may also repress GABA catabolism or alleviate feedback inhibition [23]. Supporting this notion, key GABA shunt–associated genes including *GABA-T1*, *GABA-T2*, and *SSADH* were significantly upregulated (Figure 5A), indicating enhanced flux through the GABA pathway and a net accumulation that resembles stress-induced metabolic reprogramming [24]. An exception to this pattern was observed in the *gad2* sg2 line, which failed to show a significant increase in GABA levels despite confirmed editing. Deep sequencing revealed that sgRNA2 targeted a region near the mid-to-distal portion of the CaMBD, frequently generating in-frame mutations or partial truncations that preserved much of the calmodulin-binding motif (Figure 1B). Consequently, the resulting GAD protein likely retained partial sensitivity to calmodulin, thereby limiting enzymatic derepression. This interpretation aligns with previous findings indicating that full functional release requires truncation beyond the core CaM interaction domain [18,19]. Transcriptome profiling revealed that *gad2* #1–5 lines underwent broader transcriptional reprogramming compared to *gad3* #3–8 (Figure 4A), with 1383 and 808 differentially expressed genes (DEGs), respectively. Among them, 434 DEGs were commonly altered in both lines (Figure 4B), predominantly enriched in GABA metabolism and stress response categories. Upregulation of genes encoding GADs, amino acid transporters, and ion channel components such as *KAT1* was accompanied by suppression of *GORK* and *PBP1* (Figure 4D, Table 1), consistent with a role for GABA in modulating membrane transport and osmotic homeostasis [10,25,26]. Furthermore, canonical stress-responsive transcription factors—including *WRKY46*, *ERF*, and *NAC*—were transcriptionally suppressed in the edited lines, as confirmed by both RNA-seq (Table 1) and qRT-PCR analyses (Figure 5B). This repression may reflect the attenuation of classical abiotic stress signaling pathways under conditions of constitutive GABA accumulation. This hypothesis is supported by earlier studies reporting that exogenous GABA application can suppress ABA-mediated gene expression and reactive oxygen species production during stress exposure [27,28]. Collectively, these findings suggest that CaMBD-truncated GADs not only elevate GABA levels but also orchestrate a systemic shift in transcriptional stress adaptation. Interestingly, while GABA levels increased significantly, FAA pools remained largely unchanged (Figure 3B). In BR+10 fruits, FAA concentrations were comparable to WT, even under conditions of elevated GABA. This selective redirection of glutamate into the GABA pathway, without disrupting overall nitrogen metabolism, contrasts with broader amino acid remodeling typically observed under drought or salinity stress [29,30]. Such metabolic precision is particularly valuable in the context of functional food development, where trait enhancement must avoid compromising core nutritional quality.

Taken together, our findings demonstrate that CRISPR/Cas9-mediated truncation of the CaM-binding domains in *SlGAD2* and *SlGAD3* effectively relieves calmodulin-dependent inhibition, leading to sustained GABA overaccumulation without perturbing global amino acid homeostasis. Among the edited lines, *SlGAD2* sg1 exhibited the most pronounced phenotypic and transcriptomic changes, suggesting a dominant role for *SlGAD2* in coordinating GABA-associated developmental and stress adaptation processes. By contrast, the *gad2* sg2 line, which retained partial CaMBD functionality, failed to induce GABA hyperaccumulation, reinforcing the critical importance of complete CaMBD disruption for functional depression. These results collectively support a model in which engineered GAD enzymes can fine-tune GABA biosynthesis and reprogram stress-related transcriptional networks in a domain-specific manner. This strategy offers a promising metabolic engineering approach for generating GABA-enriched functional crops while maintaining growth and compositional integrity—an essential consideration for nutritional improvement and consumer acceptance in horticultural biotechnology.

## 5. Conclusions

This study demonstrates that CRISPR/Cas9-mediated editing of *SlGAD2* and *SlGAD3*, specifically targeting the C-terminal calmodulin-binding domain (CaMBD), effectively enhances GABA accumulation in tomato fruit. The resulting truncated GAD proteins lack the inhibitory CaMBD, leading to constitutive GAD activity and sustained GABA biosynthesis. Among the two genes, *SlGAD2* editing exerted a stronger impact, inducing distinct vegetative phenotypes and broader transcriptomic shifts, suggesting a more dominant regulatory role compared to *SlGAD3*. Transcriptome profiling revealed that both *SlGAD2* and *SlGAD3* mutants exhibited a shared set of differentially expressed genes related to the GABA shunt and amino acid transport, while also downregulating classical abiotic stress-responsive transcription factors such as *WRKY*, *ERF*, and *NAC*. These changes support the dual role of GABA as a metabolic intermediate and a signaling regulator that fine-tunes gene expression under developmental and possibly stress-related conditions. Importantly, total free amino acid levels and fruit morphology remained largely unaffected, indicating that targeted GABA enhancement was achieved without disrupting nitrogen balance or growth traits. These findings highlight the potential of CaMBD truncation in GADs as a precise and minimally disruptive strategy for metabolic engineering. Our work provides a mechanistic basis for GABA enrichment through genome editing and offers a foundation for developing functional tomato cultivars with enhanced nutraceutical value. The edited lines also represent a useful resource for investigating GABA-mediated transcriptional and physiological regulation during fruit development.

## Figures and Tables

**Table 1:** Representative key DEGs commonly altered in WT vs. *gad2* #1–5 and *gad3* #3–8.

Category	Gene ID	Gene Description	WT vs. gad2 #1–5		WT vs. *gad3* #3–8	
			log_2_FC	padj	log_2_FC	padj
GABA Shunt	Solyc01g005000.3.1	Glutamate decarboxylase (GAD)	1.31	9 × 10^−4^	1.73	6 × 10^−6^
	Solyc01g080280.3.1	Chloroplast glutamine synthetase	2.92	2 × 10^−40^	1.35	1 × 10^−8^
	Solyc03g078150.3.1	Amino acid transporter family protein	2.52	2 × 10^−27^	1.22	2 × 10^−6^
	Solyc03g113980.3.1	Calmodulin binding protein-like	−2.69	5 × 10^−5^	−1.93	6 × 10^−3^
	Solyc03g120090.1.1	Pyridoxal 5′-phosphate synthase pdxS subunit	−1.94	6 × 10^−103^	−1.47	3 × 10^−62^
Cell membrane transport and Ca^2+^-K^+^ ion transportation	Solyc10g006660.3.1	Calcium-binding protein PBP1	−1.16	3 × 10^−8^	−2.3	4 × 10^−27^
	Solyc09g007860.4.1	Calcium-dependent lipid-binding (CaLB domain) protein	−1.23	1 × 10^−16^	−1.23	3 × 10^−16^
	Solyc02g091500.1.1	Calcium-dependent protein kinase (CDPK)	−1.58	7 × 10^−4^	−1.43	4 × 10^−3^
	Solyc05g051220.3.1	Potassium outward rectifying channel GORK	−1.02	3 × 10^−9^	−1.45	9 × 10^−17^
	Solyc08g016500.3.1	Potassium inward channel KAT1	1.06	2 × 10^−3^	1.13	1 × 10^−3^
	Solyc10g006800.4.1	Cyclic nucleotide-gated ion channel 4	−1.93	2 × 10^−4^	−2.42	4 × 10^−6^
	Solyc02g094000.1.1	EF-hand domain protein (Ca^2+^ binding)	−2.19	5 × 10^−5^	−3.37	3 × 10^−9^
	Solyc03g026280.3.1	C-repeat binding factor 1 (possibly involved in cold/ion stress)	−3.03	2 × 10^−6^	−5.03	5 × 10^−12^
	Solyc09g005260.4.1	Vacuolar cation/proton exchanger (ion homeostasis under stress)	−2.14	5 × 10^−32^	−2.15	5 × 10^−32^
Stress Response	Solyc01g098270.1.1	Chaperone protein DnaJ	−6.16	3 × 10^−21^	−7.35	1 × 10^−22^
	Solyc09g092260.4.1	Chaperone protein DnaJ	1.74	8 × 10^−5^	2.33	5 × 10^−8^
	Solyc09g005120.3.1	DnaJ protein ERDJ3A	−2.25	7 × 10^−138^	−1.32	8 × 10^−54^
	Solyc02g077670.3.1	DnaJ-like protein	−1.85	1 × 10^−232^	−1.26	5 × 10^−117^
	Solyc04g007470.3.1	Drought responsive Zinc finger protein	1.28	2 × 10^−4^	1.34	1 × 10^−4^
	Solyc02g077610.3.1	NAC domain protein	−2.07	3 × 10^−44^	−1.06	3 × 10^−13^
	Solyc02g093420.4.1	NAC domain-containing protein 10	−1.37	1 × 10^−6^	−1.13	1 × 10^−4^
	Solyc09g015770.3.1	WRKY transcription factor 81	−1.12	2 × 10^−8^	−1.91	8 × 10^−21^
	Solyc04g077980.1.1	C2H2-type zinc finger protein	−1.58	4 × 10^−10^	−2.98	3 × 10^−29^
	Solyc10g006130.1.1	EAR motif-containing protein *SlERF36*	−1.33	1 × 10^−11^	−1.94	2 × 10^−22^

## Data Availability

The original contributions presented in the study are included in the article/Supplementary Materials; further inquiries can be directed to the corresponding authors.

## Supplementary Materials

The following supporting information can be downloaded at https://www.mdpi.com/article/10.3390/genes16070744/s1, Figure S1. Generation and molecular selection of CRISPR/Cas9-edited tomato transgenic. Figure S2. PCR-based identification of null segregants lacking the transgene in *SlGAD2* and *SlGAD3* edited lines. Figure S3. Transcriptome profiling of *gad2* and *gad3* mutant tomato fruits compared to the wild type. Table S1. Designed sgRNA of *SlGAD2* and *SlGAD3* in tomato genome using CRISPR RGEN tools “http://www.rgenome.net/ (accessed on 20 April 2022)”. Table S2. List of primers used in this study. Table S3. *SlGAD* transgenic plants and Genotype ratio generated using the CRISPR/Cas9 system. Table S4. Statistics of reads mapping to reference (ITAG4.0). Table S5. List of common DEGs between WT and *SlGAD* lines.
