# Supplementary material for: Transcriptomic Insights into GABA Accumulation in Tomato via CRISPR/Cas9-Based Editing of SlGAD2 and SlGAD3
Source: Genes (Basel). 2025 Jun 26;16(7):744. doi: 10.3390/genes16070744 (PMC12294585; doi:10.3390/genes16070744)
Supplement: Supplementary file 1 [file genes-16-00744-s001.zip › genes-3723160-supplementary.pdf]

Supplementary Data

Transcriptomic Insights into GABA Accumulation in Tomato via CRISPR/Cas9-Based Editing of *SlGAD2* and *SlGAD3*

Jin-Young Kim[1], Dong-Hyun Kim[1], Yu-Jin Jung[1,2], and Kwon Kyoo Kang [1,2*]

Contents

Supplementary Figure S1. Generation and molecular selection of CRISPR/Cas9-edited tomato lines.

Supplementary Figure S2. PCR-based identification of null segregants lacking the transgene in *SlGAD2* and *SlGAD3* edited lines.

Supplementary Figure S3. Transcriptome profiling of *gad2* and *gad3* mutant tomato fruits compared to the wild type.

Supplementary Table S1. Designed sgRNA of SlGAD2 and SlGAD3 in tomato genome using CRISPR RGEN tools (http://www.rgenome.net/).

Supplementary Table S2. List of primers used in this study.

Supplementary Table S3. *SlGAD* transgenic plants and Genotype ratio generated using the CRISPR/Cas9 system.

Supplementary Table S4. Statistics of reads mapping to reference (ITAG4.0)

Supplementary Table S5. List of common DEGs between WT and *SlGAD* lines.

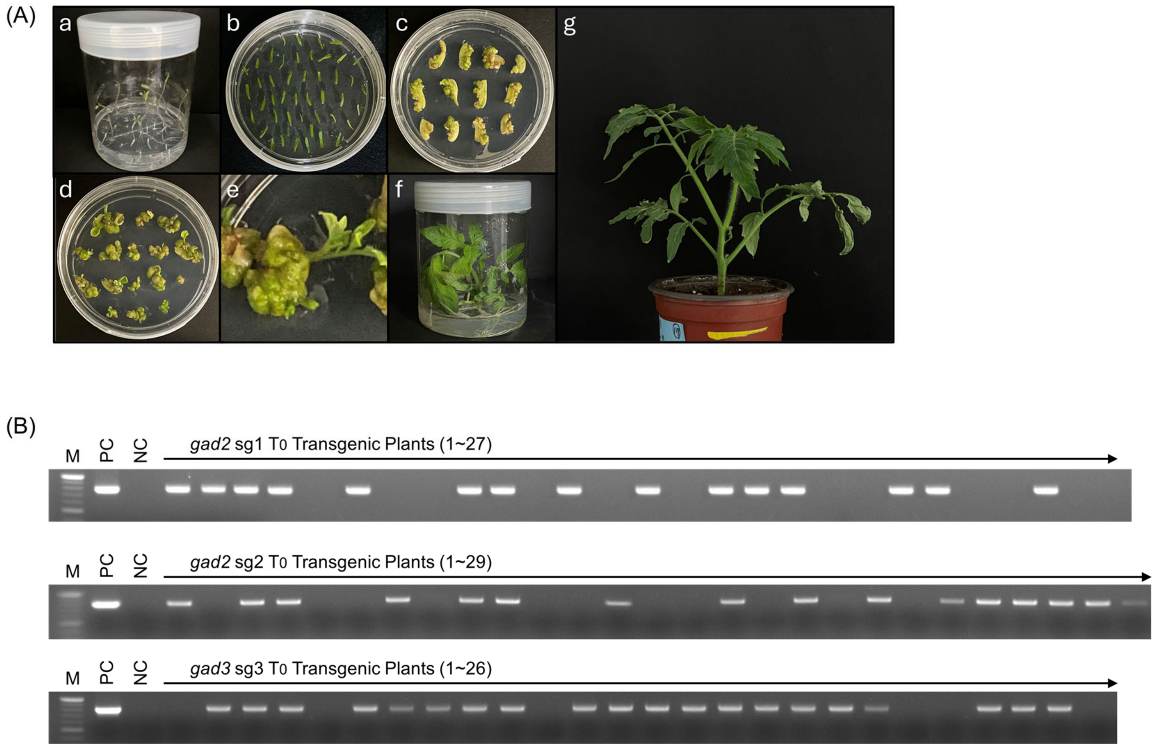

Supplementary Figure S1. Generation and molecular selection of CRISPR/Cas9-edited tomato lines. (A) Workflow of transgenic plant production via *Agrobacterium tumefaciens*-mediated transformation. (a) Germinated seedlings used as source of explants; (b) Infection of cotyledon explants with *Agrobacterium tumefaciens* harboring the CRISPR/Cas9 construct; (c) Callus induction from infected explants; (d) Shoot regeneration from proliferating calli; (e) Elongation of regenerated shoots; (f) Root induction in elongated shoots; (g) Acclimatized transgenic plant established in soil under greenhouse conditions. (B) PCR screening of putative Transgenic plants using NPTII-specific primers to verify the insertion of the T-DNA region. Amplification in T_0 lines indicates successful transformation. M: DNA marker; PC: positive control; NC: negative control(wild-type).

(A) *gad2 #1-5*

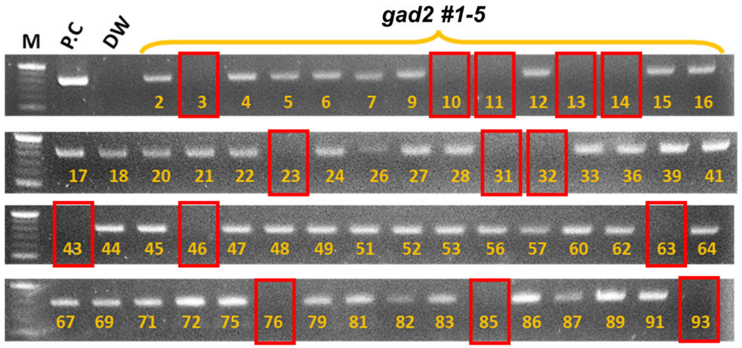

(B) *gad3 #3-8*

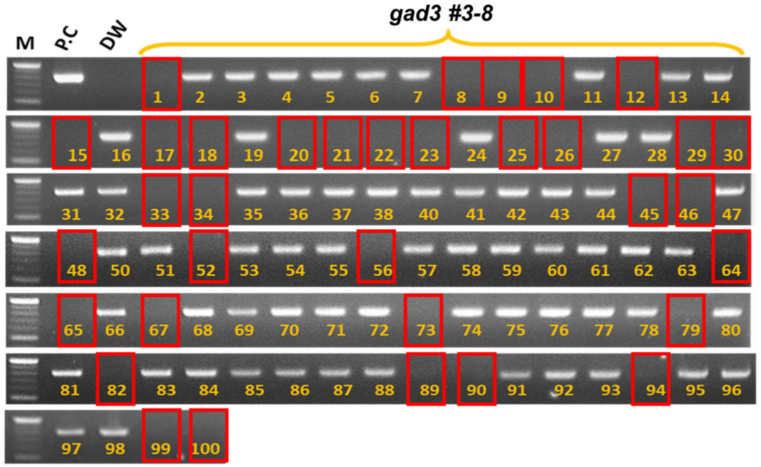

Supplementary Figure S2. PCR-based identification of null segregants lacking the transgene in *SlGAD2* and *SlGAD3* edited lines. PCR analysis was performed using NPTII-specific primers to detect the presence or absence of the T-DNA in T_2 progenies derived from *gad2 #1–5* and *gad3 #3–8* lines. Individuals showing no amplification were considered null segregants, indicating loss of the transgene through segregation. Red boxes indicate PCR-negative individuals (null segregants). M: DNA marker; PC: positive control

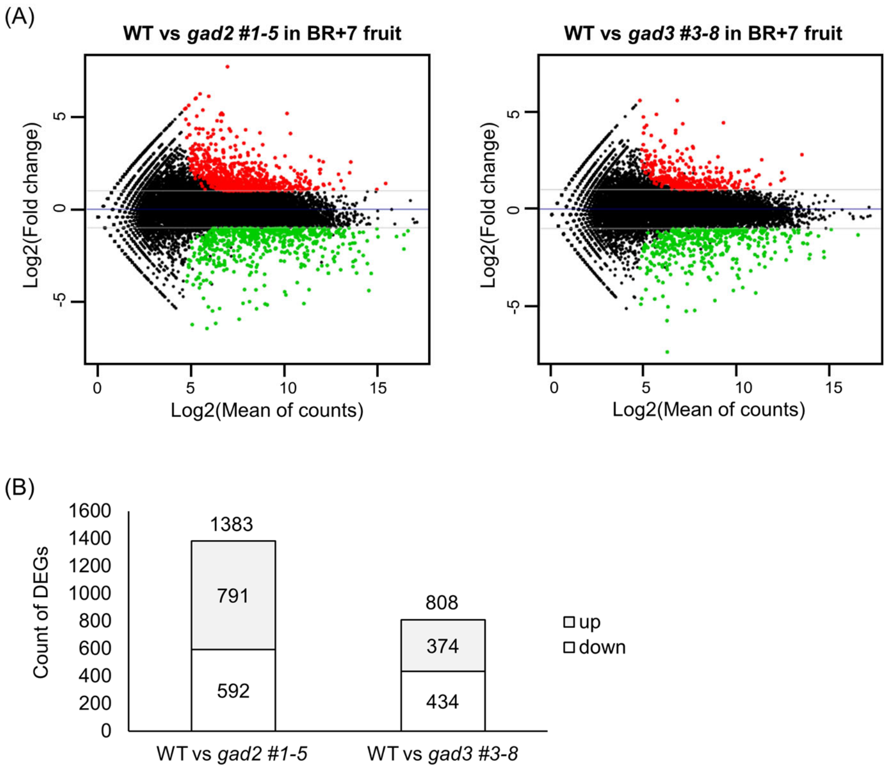

Supplementary Figure S3. Transcriptome profiling of *gad2* and *gad3* mutant tomato fruits compared to the wild type. (A) MA plots showing differentially expressed genes (DEGs) in red ripe fruits of *gad2 #1–5* and *gad3 #3–8* compared to wild type. Each dot represents a gene; red and green dots indicate significantly upregulated and downregulated genes, respectively ($|\log_2 FC| > 1$, adjusted $p < 0.05$). (B) Summary of DEG counts. A total of 1,383 DEGs (791 upregulated, 592 downregulated) were detected in *gad2 #1–5* vs. WT, and 808 DEGs (374 upregulated, 434 downregulated) were identified in *gad3 #3–8* vs. WT.

Supplementary Table S1. Designed sgRNA of SlGAD2 and SlGAD3 in tomato genome using CRISPR RGEN tools (http://www.rgenome.net/).

Gene	sgRNA Target (5' to 3')	Direction	GC Contents (%, w/o PAM)	Out-of-frame Score	Mismatches		
					0	1	2
GAD2	TGTCAAAGCTAAGCTCGCCGTGG	+	55.0	53.9	1	0	0
	CAGCGTGGCACATCATAAAACGG	+	45.0	61.0	1	0	0
GAD3	CCCGAATGCCAAAAAAGTGGAGG	+	50	57.3	1	0	0

Supplementary Table S2. List of primers used in this study.

primer	sequence (5' -3')	purpose
SlGAD2 sgRNA1 up	gattTGTCAAAGCTAAGCTCGCCG	
SlGAD2 sgRNA1 down	aaacCGGCGAGCTTAGCTTTGACA	
SlGAD2 sgRNA2 up	gattCAGCGTGGCACATCATAAAA	sgRNA anealing
SlGAD2 sgRNA2 down	aaacTTTTATGATGTGCCACGCTG	
SlGAD3 sgRNA3 up	gattCCCGAATGCCAAAAAAGTGG	
SlGAD3 sgRNA3 down	aaacCCACTTTTTTGGCATTCGGG	
Kanamycin-R Fw	ATGATTGAACAAGATGGATTGCAC	transgenic selection PCR
Kanamycin-R Rv	TCAGAAGAACTCGTCAAGAAGGC	
1st NGS PCR (*SlGAD2* sg1,2) Fw	TTTAGAGTGTTACTACATGTTTGATGG	
1st NGS PCR (*SlGAD2* sg1,2) Rv	GCCTTTAAAATGATGGTCCAA	
2nd NGS PCR (*SlGAD2* sg1) Fw	acactctttccctacacgacgctcttccgatctcCCTAATTTTTATATTTTTCGTTCGTTT	
2nd NGS PCR (*SlGAD2* sg1) Rv	gtgactggagttcagacgtgtgctcttccgatctGCAACAAACTTCTTCCATGCT	
2nd NGS PCR (*SlGAD2* sg2) Fw	acactctttccctacacgacgctcttccgatctcTTTTGAAATAGGTTATGGACATCG	deep-sequnecing anlysis
2nd NGS PCR (*SlGAD2* sg2) Rv	gtgactggagttcagacgtgtgctcttccgatctTGAATTAATTAACAAACTCCCATAGTC	
1st NGS PCR (*SlGAD3* sg3) Fw	CGTGAAAATGCAATTGTGCT	
1st NGS PCR (*SlGAD3* sg3) Rv	CAACCATTAATCCTTCCCTAACA	
2nd NGS PCR (*SlGAD3* sg3) Fw	acactctttccctacacgacgctcttccgatctcTAGGTTTGGGTGGATTGTCC	
2nd NGS PCR (*SlGAD3* sg3) Rv	gtgactggagttcagacgtgtgctcttccgatctCATATTTCTTCCAAAACTCAGCAA	
SlACTIN Fw	GGGATGGAGAAGTTTGGTGGTGG	
SlACTIN Rv	CTTCGACCAAGGGATGGTGTAGC	
SlGDH Fw	AGCTGATGAGGTCAGTGCAA	
SlGDH Rv	CGCAGACCTAGAGGCTGTTT	qRT-PCR anlysis
SlGAD2 Fw	ACACTATGCCACCAGATGCT	
SlGAD2 Rv	GCTTTGACACGTGCTGGAA	
SlGAD3 Fw	AGACAATAGCCTCCACAACGA	
SlGAD3 Rv	CCTCTCTAATCACAACGCGC	

SlGABA-T1 Fw	GGGGACTTGGTTTGATCCTT
SlGABA-T1 Rv	TCACCAGCAACTCGTACCAA
SlGABA-T2 Fw	GGTTGCATTGGAAGCACTAAA
SlGABA-T2 Rv	CCAGTTCCCCTTATCTCTCCA
SlGABA-T3 Fw	AGGGGAACTGGTTTGCTACA
SlGABA-T3 Rv	CCAACACTCCATGCTTCTCA
SlSSADH Fw	AGGGAGCCAAAGTCCTTGTT
SlSSADH Rv	ACTTCAAAAGAGGGGCGACT
SlWRKY46 Fw	TCACCAAAGATGCCAAGAGAA
SlWRKY46 Rv	TTGACTGGACATGATGGTGC
SlNAC Fw	AGAGAACGATGCATGGAGGT
SlNAC Rv	TCAGGAAATTGGCAATGGAGC
SlERF Fw	TCACCCGAGCCATTAACAAC
SlERF Rv	ACCTTTGCTCTTTTCTCACATTT

Supplementary Table S3. *SlGAD* transgenic plants and Genotype ratio generated using the CRISPR/Cas9 system.

Gene		No. of Regeneration plants	No. of Transgenic plants	No. of Edited plants	Genotype			
					Homo	Hetero	Bi-allelic	Multiple
GAD2	sg1	27	15	8	2	5	0	1
	sg2	29	16	9	2	2	2	3
GAD3	sg3	26	20	17	1	4	6	6

Supplementary Table S4. Statistics of reads mapping to reference (ITAG4.0)

Description	Total reads	Aligned 0 times		Aligned exactly 1 time		Aligned > 1 times		Mapping rate	
		Reads(ea)	Percent(%)	Reads(ea)	Percent(%)	Reads(ea)	Percent(%)	Reads(ea)	Percent(%)
WT (1)	10,241,249	362,552	3.54%	9,577,596	93.52%	301,101	2.94%	9,878,697	96.46%
WT (2)	10,241,249	317,818	3.10%	9,597,158	93.71%	326,273	3.19%	9,923,431	96.90%
WT (3)	10,241,249	329,973	3.22%	9,588,484	93.66%	319,546	3.01%	9,894,546	96.88%
gad2 #1-5 (1)	11,542,081	401,558	3.48%	10,867,133	94.15%	273,390	2.37%	11,140,523	96.52%
gad2 #1-5 (2)	11,542,081	365,650	3.17%	10,873,504	94.21%	302,927	2.67%	11,176,431	96.83%
gad2 #1-5 (3)	11,542,081	388,586	3.35%	10,871,513	94.18%	294,419	2.48%	11,168,490	96.71%
gad3 #3-8 (1)	11,086,146	445,544	4.02%	10,324,419	93.13%	316,183	2.85%	10,640,602	95.98%
gad3 #3-8 (2)	11,086,146	375,956	3.39%	10,357,683	93.43%	352,507	3.18%	10,710,190	96.61%
gad3 #3-8 (3)	11,086,146	429,033	3.87%	10,341,877	93.24%	334,498	2.99%	10,684,239	96.18%

Supplementary Table S5. List of common DEGs between WT and *SlGAD* lines.

Gene id	WT vs *gad2* #1-5		WT vs *gad3* #3-8		Description
	log2FoldChange	padj	log2FoldChange	padj	
Solyc01g005000.3.1	1.31	9.E-04	1.73	6.E-06	Glutamate decarboxylase (AHRD V3.3 *** B1Q3F2_SOLLC)
Solyc01g005257.1.1	1.72	2.E-05	1.58	2.E-04	Sec14p-like phosphatidylinositol transfer family protein (AHRD V3.3 *** A0A1Y1I4L4_KLENI)
Solyc01g005290.4.1	2.09	2.E-09	1.68	6.E-06	Sec14p-like phosphatidylinositol transfer family protein (AHRD V3.3 *** A0A1Y1I4L4_KLENI)
Solyc01g005300.4.1	1.68	1.E-24	1.01	7.E-09	Adagio-like protein 1 (AHRD V3.3 *** A0A1U8H9V5_CAPAN)
Solyc01g005470.3.1	1.24	4.E-03	1.58	1.E-04	protein PLANT CADMIUM RESISTANCE 2-like (AHRD V3.3 *** A0A1U8ETE7_CAPAN)
Solyc01g008420.3.1	3.28	1.E-05	3.32	2.E-05	Protein DETOXIFICATION (AHRD V3.3 *** S6BP63_TOBAC)
Solyc01g028807.1.1	1.19	4.E-26	1.71	9.E-55	Unknown protein
Solyc01g049880.3.1	1.39	1.E-06	1.03	9.E-04	Unknown protein
Solyc01g079470.4.1	3.50	7.E-44	1.38	6.E-07	Unknown protein
Solyc01g080280.3.1	2.92	2.E-40	1.35	1.E-08	chloroplast glutamine synthetase
Solyc01g081390.4.1	1.17	4.E-08	1.18	5.E-08	Xylulose 5-phosphate/phosphate translocator, chloroplastic (AHRD V3.3 *** A0A1U8EHP7_CAPAN)
Solyc01g086920.3.1	2.58	4.E-06	2.00	9.E-04	Leucine-rich repeat receptor-like protein (AHRD V3.3 *** H6V789_MALDO)
Solyc01g087790.2.1	2.26	4.E-04	2.47	1.E-04	Subtilisin-like protease (AHRD V3.3 *** O82006_SOLLC)
Solyc01g090680.3.1	1.85	2.E-13	1.15	2.E-05	Uncharacterized conserved protein (UCP012943) (AHRD V3.3 *** AT4G25170.1)
Solyc01g102350.3.1	1.85	1.E-05	1.98	3.E-06	Pectin acetylesterase (AHRD V3.3 *** K4B1G3_SOLLC)
Solyc01g102390.4.1	1.75	7.E-04	1.50	8.E-03	Germin-like protein (AHRD V3.3 *** O65358_SOLTU)
Solyc01g104710.3.1	1.13	1.E-06	1.34	5.E-09	thionin-like protein (AHRD V3.3 -** AT1G25275.2)
Solyc01g104950.4.1	1.71	2.E-07	2.05	2.E-10	LEXYL2
Solyc01g108513.1.1	1.48	3.E-16	2.12	3.E-33	Alpha/beta-Hydrolases superfamily protein (AHRD V3.3 *** A0A2U1KK17_ARTAN)
Solyc01g108910.4.1	1.32	2.E-13	1.03	4.E-08	maternal effect embryo arrest 14 (AHRD V3.3 *** AT2G15890.1)
Solyc01g110360.3.1	2.86	2.E-252	2.01	2.E-124	Fructose-bisphosphate aldolase (AHRD V3.3 *** Q9SXX5_NICPA)

Solyc02g005110.3.1	1.94	3.E-06	1.42	2.E-03	MADS-box protein SOC1 (AHRD V3.3 --* XP_025887600.1)
Solyc02g005606.1.1	4.14	8.E-50	1.55	8.E-07	serine/threonine-protein phosphatase 7 long form homolog (AHRD V3.3 *-* XP_019069006.1)
Solyc02g022890.3.1	2.24	2.E-05	2.35	1.E-05	Fatty acid hydroxylase (AHRD V3.3 *-* A0A200Q3T8_9MAGN)
Solyc02g022900.3.1	2.00	5.E-03	2.38	6.E-04	Unknown protein
Solyc02g055440.4.1	3.83	1.E-16	3.27	5.E-12	E3 ubiquitin-protein ligase%2C putative (DUF177) (AHRD V3.3 *** AT3G19800.2)
Solyc02g065765.1.1	1.14	4.E-09	1.48	6.E-15	COBRA-like protein (AHRD V3.3 *** K4B708_SOLLC)
Solyc02g069290.3.1	2.05	2.E-16	1.42	7.E-08	RING-type E3 ubiquitin transferase (AHRD V3.3 *-* A0A218W8P0_PUNGR)
Solyc02g070540.3.1	1.60	3.E-09	1.61	3.E-09	DUF642 domain-containing protein (AHRD V3.3 *** A0A1Q3DKA7_CEPFO)
Solyc02g070800.2.1	2.89	2.E-89	1.93	4.E-40	DUF561 domain-containing protein (AHRD V3.3 *** A0A1Q3DH97_CEPFO)
Solyc02g071050.4.1	2.74	1.E-03	2.99	4.E-04	Purine permease (AHRD V3.3 *** A0A2P5DL88_TREOI),Pfam:PF16913
Solyc02g072440.4.1	1.60	2.E-03	1.64	2.E-03	Receptor-like protein kinase (AHRD V3.3 *** A0A2R6Q6W2_ACTCH)
Solyc02g076680.3.1	2.02	2.E-08	1.28	2.E-03	DUF688 domain-containing protein (AHRD V3.3 *** A0A1Q3BIV2_CEPFO)
Solyc02g076690.3.1	3.58	3.E-27	2.48	3.E-13	Cysteine protease CP14 (AHRD V3.3 *** T2BRA8_TOBAC)
Solyc02g078670.3.1	1.29	6.E-16	1.90	2.E-34	COP1-interacting protein 7 (AHRD V3.3 *** CIP7_ARATH)
Solyc02g082440.2.1	1.45	1.E-10	1.25	7.E-08	Unknown protein
Solyc02g084720.3.1	1.12	2.E-06	1.20	6.E-07	beta-galactosidase 6
Solyc02g085760.2.1	1.26	9.E-04	1.54	4.E-05	Rhomboid domain-containing protein (AHRD V3.3 *** A0A1Q3C608_CEPFO)
Solyc02g087400.1.1	1.22	3.E-06	1.02	2.E-04	Ankyrin repeat-containing protein (AHRD V3.3 *** A0A2U1KW09_ARTAN)
Solyc02g088390.4.1	1.07	2.E-03	2.31	9.E-14	Lamin-like protein (AHRD V3.3 *** A0A2G2V9B8_CAPBA)
Solyc02g089880.4.1	2.00	4.E-03	2.24	1.E-03	Hexosyltransferase (AHRD V3.3 *** A0A1U8G2N1_CAPAN)
Solyc02g091140.3.1	1.01	7.E-04	1.16	1.E-04	S-adenosyl-L-methionine-dependent methyltransferases superfamily protein (AHRD V3.3 *** A0A1P8BFJ6_ARATH)
Solyc02g093980.3.1	1.52	7.E-04	1.55	7.E-04	Nucleolar-like protein (AHRD V3.3 *-* F4J3H8_ARATH)
Solyc03g006250.2.1	1.97	7.E-03	2.18	3.E-03	GDSL esterase/lipase (AHRD V3.3 *** A0A1U8GGV5_CAPAN)
Solyc03g006330.4.1	1.33	3.E-05	1.09	1.E-03	Leucine-rich repeat receptor-like serine/threonine-protein kinase (AHRD V3.3 *** A0A0A7LUM7_ALBBR)
Solyc03g006430.3.1	1.17	3.E-03	1.33	9.E-04	DUF616 domain-containing protein (AHRD V3.3 *** A0A1Q3BIE2_CEPFO)
Solyc03g007230.4.1	1.36	4.E-35	1.91	2.E-69	Protein phosphatase 2C (AHRD V3.3 *** A0A2U1KYU0_ARTAN)

Solyc03g013240.4.1	1.91	2.E-15	1.03	1.E-04	lysine-tRNA ligase (AHRD V3.3 *** AT3G01060.1)
Solyc03g019650.3.1	1.30	2.E-42	1.28	4.E-41	Major facilitator superfamily protein (AHRD V3.3 *-* A0A2U1MHC8_ARTAN)
Solyc03g019790.3.1	2.18	2.E-06	2.00	3.E-05	alpha-galactosidase
Solyc03g019890.3.1	1.76	1.E-08	1.84	2.E-09	beta-galactosidase 7
Solyc03g044790.3.1	1.66	1.E-03	1.45	8.E-03	methylesterase AY455313
Solyc03g053085.1.1	2.19	8.E-08	1.48	1.E-03	Ribonuclease 3-like protein 2 (AHRD V3.3 *-* A0A2G3BC36_CAPCH)
Solyc03g078150.3.1	2.52	2.E-27	1.22	2.E-06	Amino acid transporter family protein (AHRD V3.3 *** S8E0T7_9LAMI)
Solyc03g078610.3.1	1.00	2.E-03	1.02	2.E-03	Alpha-ketoglutarate-dependent dioxygenase alkB-like protein 2 (AHRD V3.3 *** A0A2G2X511_CAPBA)
Solyc03g082540.4.1	1.37	3.E-06	1.17	1.E-04	plasminogen activator inhibitor (AHRD V3.3 *-* AT1G57680.3)
Solyc03g083100.3.1	1.12	6.E-10	1.25	5.E-12	SUN-like protein 10
Solyc03g083570.3.1	1.54	8.E-06	1.16	2.E-03	Protein LOW PSII ACCUMULATION 2, chloroplastic (AHRD V3.3 *** A0A2G3CVQ8_CAPCH)
Solyc03g095310.3.1	2.72	5.E-07	2.35	3.E-05	Cytochrome P450 (AHRD V3.3 *** A0A2U1PRC1_ARTAN)
Solyc03g095750.2.1	2.98	9.E-11	2.72	9.E-09	Chlorophyllide a oxygenase (AHRD V3.3 *-* F8SPG4_CAMSI)
Solyc03g098480.1.1	3.36	5.E-15	1.55	2.E-03	chromosome alignment-maintaining phosphoprotein 1-like isoform X2 (AHRD V3.3 --* XP_017702391.1)
Solyc03g098795.1.1	2.78	5.E-09	3.91	1.E-17	Proteinase inhibitor type-2 (AHRD V3.3 *** A0A2G2XU31_CAPAN)
Solyc03g112760.3.1	3.26	2.E-29	1.38	2.E-05	transmembrane protein (AHRD V3.3 *** AT3G48200.1)
Solyc03g114730.3.1	1.75	2.E-04	2.42	1.E-07	O-fucosyltransferase (AHRD V3.3 *** A0A2P5EBU6_TREOI)
Solyc03g114970.3.1	1.60	3.E-27	1.42	3.E-21	Protein SPIRAL1 (AHRD V3.3 *** A0A2G3A261_CAPAN)
Solyc03g118510.3.1	1.80	2.E-04	1.91	1.E-04	Ovary receptor kinase 27 (AHRD V3.3 *** S4WIQ7_SOLCH)
Solyc03g120420.3.1	1.84	5.E-44	2.10	2.E-57	Unknown protein
Solyc03g120910.4.1	1.12	3.E-04	1.57	1.E-07	Homeobox leucine-zipper protein (AHRD V3.3 *** Q76CL1_ZINVI)
Solyc04g007470.3.1	1.28	2.E-04	1.34	1.E-04	Drought responsive Zinc finger protein
Solyc04g008730.3.1	2.17	7.E-19	1.56	8.E-10	Alpha-galactosidase (AHRD V3.3 *** K4BP29_SOLLC)
Solyc04g009860.4.1	1.15	8.E-03	1.24	6.E-03	2-oxoglutarate (2OG) and Fe(II)-dependent oxygenase superfamily protein (AHRD V3.3 *** A0A2U1QA13_ARTAN)
Solyc04g014830.3.1	2.62	5.E-08	1.93	2.E-04	Transcription factor GRAS (AHRD V3.3 *** A0A200QXB8_9MAGN)
Solyc04g040190.1.1	1.19	1.E-03	1.42	9.E-05	lycopene beta-cyclase,Pfam:PF05834

Solyc04g050170.4.1	4.02	2.E-07	4.20	6.E-08	Leucine-rich receptor-like protein kinase family protein (AHRD V3.3 *** A0A061F8K0_THECC)
Solyc04g050620.3.1	2.94	5.E-05	2.11	9.E-03	Cytochrome (AHRD V3.3 *** A0A2G2Y725_CAPAN)
Solyc04g054260.4.1	2.27	6.E-06	1.57	6.E-03	Cytochrome p450 (AHRD V3.3 *** A0A2K3NDQ9_TRIPR)
Solyc04g054370.1.1	1.51	8.E-03	1.69	3.E-03	RING/U-box superfamily protein (AHRD V3.3 *-* Q3EAE6_ARATH)
Solyc04g070980.4.1	1.54	9.E-07	1.59	5.E-07	cycloartenol synthase 1
Solyc04g077020.3.1	1.19	7.E-81	1.28	1.E-93	Tubulin alpha chain (AHRD V3.3 *** A0A1U8GD16_CAPAN)
Solyc04g077670.3.1	2.23	4.E-16	1.11	3.E-04	Serine carboxypeptidase-like 18 (AHRD V3.3 *** A0A2G3CPL7_CAPCH)
Solyc04g077780.3.1	1.59	2.E-20	1.19	2.E-11	LIM domain-containing protein (AHRD V3.3 *** A0A1Q3ALK2_CEPFO)
Solyc04g078520.3.1	2.67	5.E-08	2.36	3.E-06	Transcription factor MYC/MYB domain-containing protein (AHRD V3.3 *** A0A2U1MUV3_ARTAN)
Solyc05g007500.4.1	1.54	6.E-06	1.18	1.E-03	CAAX amino terminal protease family protein (AHRD V3.3 *** A0A2U1P5P7_ARTAN)
Solyc05g007780.3.1	3.54	4.E-09	2.79	1.E-05	Photosynthetic NDH subcomplex L 2 (AHRD V3.3 *** A0A0F7GYP0_9ROSI)
Solyc05g009070.4.1	1.07	8.E-07	1.93	3.E-21	Protein phosphatase 2C family protein (AHRD V3.3 *** A0A2U1KGE8_ARTAN)
Solyc05g014000.4.1	2.38	4.E-35	1.91	1.E-22	Pectate lyase (AHRD V3.3 *** A0A2G3B8Z4_CAPCH)
Solyc05g014190.4.1	2.73	7.E-08	1.78	2.E-03	CAAX amino terminal protease family protein (AHRD V3.3 *** AT3G26085.2)
Solyc05g018510.3.1	1.30	2.E-04	1.21	1.E-03	ABC transporter-like (AHRD V3.3 *** A0A200PN89_9MAGN)
Solyc05g052030.1.1	1.25	4.E-05	1.16	2.E-04	ethylene response factor 4
Solyc05g052700.4.1	2.27	1.E-05	2.13	9.E-05	Tetratricopeptide repeat (TPR)-like superfamily protein (AHRD V3.3 *** Q9SD65_ARATH)
Solyc05g053100.3.1	2.49	2.E-17	1.58	4.E-07	Dihydrolipoyl dehydrogenase-like protein (AHRD V3.3 *** A0A2K3PDU3_TRIPR)
Solyc05g053930.4.1	2.24	8.E-09	2.85	7.E-14	Protein kinase APK1B, chloroplastic (AHRD V3.3 *** A0A1U8E7T5_CAPAN)
Solyc05g055550.3.1	2.40	1.E-09	1.47	8.E-04	vacuolar acid trehalase (AHRD V3.3 *** AT3G10405.1)
Solyc06g005710.3.1	1.29	4.E-04	1.26	8.E-04	Protein TIC 62, chloroplastic (AHRD V3.3 *** A0A1J3HBE9_NOCCA)
Solyc06g005950.3.1	1.36	1.E-24	1.08	2.E-15	ATP-dependent zinc metalloprotease FTSH 7, chloroplastic (AHRD V3.3 *** A0A2G2Z858_CAPAN)
Solyc06g009190.4.1	4.30	2.E-34	2.68	3.E-13	Pectinesterase (AHRD V3.3 *** A0A2G3C3P4_CAPCH)
Solyc06g019200.4.1	2.52	8.E-19	1.40	7.E-06	Ethylene-dependent gravitropism-deficient and yellow-green-like 2 (AHRD V3.3 *** F4K0T6_ARATH)

Solyc06g033850.3.1	3.11	3.E-13	2.41	6.E-08	Dehydration-responsive element-binding protein 2d-like (AHRD V3.3 *-* A0A2K3MMA2_TRIPR)
Solyc06g036100.3.1	2.37	4.E-04	2.29	9.E-04	Transporter arsB (AHRD V3.3 *** A0A2U1Q5Y3_ARTAN)
Solyc06g051940.4.1	1.06	1.E-05	1.24	2.E-07	Protein phosphatase 2C (AHRD V3.3 *** A0A2U1KYU0_ARTAN)
Solyc06g064840.4.1	3.33	2.E-07	2.58	2.E-04	AGAMOUS-like MADS-box transcription factor (AHRD V3.3 *** K7WPA8_NARBU)
Solyc06g065690.3.1	1.19	2.E-04	1.18	4.E-04	Serine/threonine protein phosphatase 2A regulatory subunit (AHRD V3.3 *** A0A2G2WKL6_CAPBA)
Solyc06g071670.1.1	1.14	2.E-03	1.15	2.E-03	S-adenosyl-L-methionine-dependent methyltransferases superfamily protein (AHRD V3.3 *** Q9FML3_ARATH)
Solyc06g071820.3.1	1.72	2.E-91	1.41	3.E-61	BTB/POZ and TAZ domain-containing protein 1 (AHRD V3.3 *** A0A2G3BFP5_CAPCH)
Solyc06g072710.3.1	1.92	1.E-08	1.04	9.E-03	RNA polymerase sigma factor sigA (AHRD V3.3 *** A0A2G2ZD00_CAPAN)
Solyc06g082910.3.1	1.85	3.E-05	1.73	2.E-04	Formin-like protein (AHRD V3.3 *** A0A2G3CBV0_CAPCH)
Solyc07g007755.1.1	1.55	3.E-96	1.75	6.E-123	Defensin protein (AHRD V3.3 *** B1N681_SOLPI)
Solyc07g017600.3.1	1.16	4.E-12	1.20	1.E-12	Pectinesterase (AHRD V3.3 *** A0A1U8F5L9_CAPAN)
Solyc07g022900.4.1	2.46	1.E-14	1.15	2.E-03	Chlorophyll a-b binding protein, chloroplastic (AHRD V3.3 *** A0A2G2V6L4_CAPBA)
Solyc07g043420.3.1	2.25	4.E-08	2.11	6.E-07	2-oxoglutarate-dependent dioxygenase 2
Solyc07g045440.1.1	1.63	4.E-17	1.52	9.E-15	Fasciclin-like arabinogalactan protein 2 (AHRD V3.3 *** A0A2G3BYC2_CAPCH)
Solyc07g049280.3.1	1.12	8.E-03	1.33	1.E-03	Pyrophosphate--fructose 6-phosphate 1-phosphotransferase subunit beta (AHRD V3.3 *** A0A2G2Z2T1_CAPAN)
Solyc07g051820.3.1	4.36	2.E-18	2.36	2.E-05	Cellulose synthase (AHRD V3.3 *** A0A200PYU7_9MAGN)
Solyc07g055050.3.1	1.79	2.E-24	1.23	1.E-11	ATP synthase protein I-related protein (AHRD V3.3 *** A0A2U1Q771_ARTAN)
Solyc07g056540.3.1	4.10	6.E-175	2.69	9.E-75	glycolate oxidase X92888
Solyc07g062130.3.1	1.07	1.E-02	1.33	9.E-04	trifunctional UDP-glucose 4,6-dehydratase/UDP-4-keto-6-deoxy-D-glucose 3,5-epimerase/UDP-4-keto-L-rhamnose-reductase RHM1 (AHRD V3.3 *** A0A2I4GY66_9ROSI)
Solyc07g065900.3.1	4.36	7.E-09	3.91	6.E-07	Fructose-bisphosphate aldolase (AHRD V3.3 *** A0A200Q3T9_9MAGN)
Solyc07g066150.1.1	1.44	8.E-28	1.04	2.E-14	Photosystem I reaction center subunit V, chloroplastic (AHRD V3.3 *** A0A1U8HDK9_CAPAN)
Solyc08g005560.3.1	2.02	1.E-06	1.71	1.E-04	transglutaminase family protein (AHRD V3.3 *** AT4G19160.2)

Solyc08g007240.4.1	2.11	1.E-20	2.40	2.E-26	Nudix hydrolase 8 (AHRD V3.3 *** A0A1U8EVF7_CAPAN)
Solyc08g013900.3.1	1.74	2.E-05	1.59	2.E-04	Plant regulator RWP-RK family protein (AHRD V3.3 *** G7JDS3_MEDTR)
Solyc08g014130.3.1	1.31	2.E-87	1.67	4.E-145	Isopropylmalate synthase (AHRD V3.3 *** K4CJ46_SOLLC)
Solyc08g016500.3.1	1.06	2.E-03	1.13	1.E-03	Potassium channel KAT1 (AHRD V3.3 *** A0A2G3BRN2_CAPCH)
Solyc08g016720.1.1	1.67	2.E-03	1.55	8.E-03	9-cis-epoxycarotenoid dioxygenase2
Solyc08g061630.3.1	2.32	4.E-10	1.42	6.E-04	YGGT family protein (AHRD V3.3 *** F8WLD3_CITUN)
Solyc08g063000.4.1	1.10	2.E-03	1.57	4.E-06	Nucleosome assembly protein family (AHRD V3.3 *** A9TVZ4_PHYPA)
Solyc08g063040.4.1	1.12	9.E-05	1.61	3.E-09	Zinc-finger protein
Solyc08g063090.2.1	3.51	4.E-16	4.39	4.E-25	Fatty acid desaturase (AHRD V3.3 *** A0A2U1LPJ3_ARTAN)
Solyc08g067840.3.1	2.06	6.E-15	1.20	3.E-05	Photosystem II reaction center PsbP family protein (AHRD V3.3 *** A0A0F7GYP2_9ROSI)
Solyc08g068690.1.1	1.47	2.E-04	1.68	2.E-05	Tyramine n-hydroxycinnamoyl transferase (AHRD V3.3 *** Q5D8C0_CAPAN)
Solyc08g074480.1.1	1.38	1.E-254	2.80	0.E+00	Bifunctional inhibitor/lipid-transfer protein/seed storage 2S albumin superfamily protein (AHRD V3.3 *** A0A2U1PU47_ARTAN)
Solyc08g076050.4.1	1.98	2.E-05	1.96	3.E-05	Receptor-like kinase (AHRD V3.3 *** P93068_BRAOL)
Solyc08g076060.3.1	1.43	3.E-17	1.49	2.E-18	Lectin protein kinase family protein (AHRD V3.3 *** A0A1I9LPL5_ARATH)
Solyc08g078210.3.1	1.13	2.E-03	1.31	3.E-04	Nudix hydrolase 8 (AHRD V3.3 *** A0A2G2XL67_CAPBA)
Solyc08g078460.3.1	1.28	7.E-05	1.22	3.E-04	Oxidoreductase family protein (AHRD V3.3 *** A0A2U1N6F1_ARTAN)
Solyc08g078760.1.1	2.62	1.E-06	1.69	6.E-03	transmembrane protein (AHRD V3.3 *** AT4G17250.1)
Solyc08g080830.3.1	1.01	1.E-02	1.15	3.E-03	Receptor kinase, putative (AHRD V3.3 *** B9RC93_RICCO)
Solyc08g082400.1.1	1.64	9.E-15	1.42	5.E-11	Chlororespiratory reduction31 (AHRD V3.3 *** A0A0F7GYA7_9ROSI)
Solyc08g082440.3.1	1.60	3.E-09	1.11	1.E-04	UDP-glucose 4-epimerase (AHRD V3.3 *** Q6XZA0_SOLTU)
Solyc09g007030.3.1	1.11	2.E-03	1.32	3.E-04	Kinesin-like protein (AHRD V3.3 *** A0A2G3BJP1_CAPCH)
Solyc09g009820.4.1	1.46	4.E-08	1.25	8.E-06	Thioredoxin family protein (AHRD V3.3 *** Q940I2_ARATH)
Solyc09g009940.3.1	1.38	3.E-25	1.07	4.E-15	Signal recognition particle protein (AHRD V3.3 *** A0A1U8EGH6_CAPAN)
Solyc09g010080.3.1	4.03	3.E-06	4.31	5.E-07	invertase 5
Solyc09g010430.1.1	1.11	4.E-04	1.72	5.E-09	Unknown protein
Solyc09g010860.4.1	2.06	8.E-07	2.17	2.E-07	expansin precursor 4

Solyc09g010940.4.1	1.40	1.E-16	1.52	1.E-19	Haloacid dehalogenase-like hydrolase (HAD) superfamily protein (AHRD V3.3 *** A0A1P8BF18_ARATH)
Solyc09g011380.3.1	1.44	3.E-04	1.22	4.E-03	BEL1-like homeodomain protein 9 (AHRD V3.3 *** A0A2G2VXF9_CAPBA)
Solyc09g011770.3.1	1.39	7.E-04	1.60	9.E-05	RING/FYVE/PHD zinc finger superfamily protein (AHRD V3.3 *-* B6SN54_MAIZE)
Solyc09g011810.3.1	2.79	7.E-67	1.65	3.E-23	Fructose-1,6-bisphosphatase (AHRD V3.3 *** B6TSK5_MAIZE)
Solyc09g042670.4.1	2.59	2.E-06	3.18	2.E-09	Unknown protein
Solyc09g083120.3.1	1.02	5.E-03	1.01	8.E-03	Acylamino-acid-releasing enzyme (AHRD V3.3 *** A0A1J3FLE9_NOCCA)
Solyc09g083130.3.1	2.51	1.E-22	1.55	1.E-08	Acylamino-acid-releasing enzyme (AHRD V3.3 *** A0A1J3E2A0_NOCCA)
Solyc09g083190.3.1	3.06	1.E-24	1.28	2.E-04	NAD(P)H dehydrogenase 18 (AHRD V3.3 *** A0A0F7CYQ9_9ROSI)
Solyc09g091990.3.1	1.41	2.E-05	1.03	4.E-03	Receptor like protein kinase S.2 (AHRD V3.3 *** A0A2G2ZTR0_CAPAN)
Solyc09g092260.4.1	1.74	8.E-05	2.33	5.E-08	Chaperone protein DnaJ (AHRD V3.3 *** A0A2G3AV72_CAPCH)
Solyc09g092330.3.1	1.21	2.E-10	1.23	1.E-10	UDP-glucuronate 4-epimerase 4 (AHRD V3.3 *** A0A1J3GBJ9_NOCCA)
Solyc09g092520.3.1	1.88	2.E-18	4.44	9.E-118	xyloglucan endotransglycosylase
Solyc10g009380.3.1	1.46	3.E-05	1.07	6.E-03	DUF688 domain-containing protein (AHRD V3.3 *** A0A1Q3BEA6_CEPFO)
Solyc10g018300.3.1	2.10	2.E-99	1.38	2.E-42	Transketolase (AHRD V3.3 *** A0A200R9X0_9MAGN)
Solyc10g051120.3.1	2.14	3.E-12	1.85	5.E-09	Mitochondrial pyruvate carrier (AHRD V3.3 *** A0A2G2WMA1_CAPBA)
Solyc10g054080.3.1	2.03	2.E-05	2.11	1.E-05	Kinesin-related protein 11 (AHRD V3.3 *** A0A2G3BDZ7_CAPCH)
Solyc10g076710.3.1	1.89	7.E-05	1.48	5.E-03	Phosphoinositide phospholipase C (AHRD V3.3 *** O49950_SOLTU)
Solyc10g078250.1.1	2.83	9.E-17	1.16	5.E-03	Peptidylprolyl isomerase (AHRD V3.3 *-* A0A2G2VW61_CAPBA)
Solyc10g078770.2.1	1.58	4.E-04	1.29	8.E-03	11 kDa late embryogenesis abundant protein (AHRD V3.3 *-* A0A2G3BHR7_CAPCH)
Solyc10g078920.3.1	2.31	1.E-11	1.58	1.E-05	Thioredoxin-like 3-1, chloroplastic (AHRD V3.3 *** A0A2G3BHN6_CAPCH)
Solyc10g080680.3.1	1.19	9.E-03	1.36	3.E-03	Adenylyl-sulfate kinase (AHRD V3.3 *** A0A2U1MP59_ARTAN),Pfam:PF01583
Solyc10g080940.3.1	1.46	2.E-57	1.03	3.E-28	Tubulin beta chain (AHRD V3.3 *** A0A1U8EJY6_CAPAN)
Solyc10g083670.3.1	1.74	5.E-04	1.64	2.E-03	Glucomannan 4-beta-mannosyltransferase 2 (AHRD V3.3 *** A0A2G3BGF1_CAPCH)
Solyc10g084280.2.1	1.37	2.E-06	1.58	3.E-08	SUN-like protein 29
Solyc10g084690.2.1	1.14	4.E-03	1.28	1.E-03	ADP-ribosylation factor GTPase-activating protein 1 (AHRD V3.3 *** A0A1U8EA26_CAPAN)

Solyc10g086150.2.1	1.74	1.E-44	1.17	9.E-20	RNA-binding protein (AHRD V3.3 *** A0A2U1PI86_ARTAN)
Solyc11g007900.3.1	1.88	8.E-12	2.13	6.E-15	EEIG1/EHBP1 N-terminal domain (AHRD V3.3 *-* A0A200R5E0_9MAGN)
Solyc11g010350.2.1	2.58	9.E-06	1.86	4.E-03	'putative glucan 1,3-beta-glucosidase (AHRD V3.3 *** Q5W708_ORYSJ)
Solyc11g012670.1.1	1.39	4.E-10	1.01	3.E-05	Pentatricopeptide repeat-containing protein (AHRD V3.3 *** A0A2U1LWS3_ARTAN)
Solyc11g013330.3.1	1.06	5.E-06	1.12	2.E-06	BPS1-like protein (AHRD V3.3 *** AT1G22030.1)
Solyc11g020960.2.1	3.45	7.E-17	1.33	8.E-03	Proteinase inhibitor type-2 (AHRD V3.3 *** A0A2G3AT14_CAPCH)
Solyc11g021360.3.1	1.61	1.E-10	1.40	5.E-08	Unknown protein
Solyc11g045110.3.1	2.76	9.E-05	2.47	1.E-03	Sucrose-phosphate synthase family protein (AHRD V3.3 *** A0A2U1M1C4_ARTAN)
Solyc11g056680.1.1	1.25	1.E-08	2.16	6.E-25	Leucine-rich repeat receptor-like protein (AHRD V3.3 *** H6V788_MALDO)
Solyc11g066720.3.1	1.03	1.E-06	1.59	3.E-15	UDP-apiose/UDP-xylose synthase (AHRD V3.3 *** A0A1B3TP38_SPIPO)
Solyc11g069590.2.1	1.01	2.E-03	1.21	2.E-04	Receptor-like serine/threonine-protein kinase NCRK (AHRD V3.3 *** A0A2G3BBL7_CAPCH)
Solyc11g071970.2.1	2.05	2.E-05	1.45	7.E-03	Serine/threonine-protein kinase Nek5 (AHRD V3.3 *** A0A2G2YGZ2_CAPAN)
Solyc11g072820.3.1	1.21	1.E-03	1.16	4.E-03	Kinesin (AHRD V3.3 *** A0A200R660_9MAGN)
Solyc11g073120.2.1	2.35	2.E-35	1.45	3.E-13	R2R3MYB transcription factor 58
Solyc12g009600.2.1	2.48	5.E-08	1.62	1.E-03	Thylakoid lumenal 16.5 kDa protein, chloroplastic (AHRD V3.3 *** A0A2G2YVM2_CAPAN)
Solyc12g011023.1.1	1.08	3.E-03	3.09	6.E-25	Xyloglucan endotransglucosylase/hydrolase (AHRD V3.3 *** Q6RHX7_SOLLC)
Solyc12g036170.2.1	3.20	6.E-09	1.98	1.E-03	Photosynthetic NDH subcomplex B 4 (AHRD V3.3 *** A0A0F7GXZ5_9ROSI)
Solyc12g055840.2.1	2.46	2.E-16	2.50	9.E-17	Glucan endo-1,3-beta-glucosidase 10 (AHRD V3.3 *** A0A1U8F6Q1_CAPAN)
Solyc12g062250.2.1	1.84	2.E-19	1.19	4.E-08	Phosphoglucan phosphatase LSF1, chloroplastic (AHRD V3.3 *** A0A1U8GMC0_CAPAN)
Solyc12g088220.2.1	1.55	1.E-89	1.24	3.E-55	SlBCAT1
Solyc12g088240.2.1	1.40	3.E-04	1.19	4.E-03	Glycosyltransferase (AHRD V3.3 *** W8Q306_ARATH)
Solyc12g099200.2.1	1.21	6.E-25	1.26	4.E-27	Invertase inhibitor (AHRD V3.3 *** O49908_TOBAC)
Solyc01g007190.3.1	-1.87	6.E-18	-1.51	4.E-12	Unknown protein
Solyc01g009770.3.1	-2.08	1.E-03	-1.90	6.E-03	ATP-dependent RNA helicase (AHRD V3.3 *** Q5HZ54_ARATH)
Solyc01g059965.1.1	-2.19	1.E-36	-1.63	9.E-22	Glucan endo-1,3-beta-glucosidase B (AHRD V3.3 *** E13B_SOLLC)

Solyc01g060020.4.1	-2.18	8.E-37	-1.62	9.E-22	beta-1,3-glucanase TOMB13GLUB
Solyc01g065530.3.1	-1.65	9.E-49	-1.25	3.E-29	COBRA-like protein (AHRD V3.3 *** A0A2G3AIT3_CAPAN)
Solyc01g066457.1.1	-4.35	3.E-53	-3.51	3.E-39	Alpha/beta-Hydrolases superfamily protein (AHRD V3.3 *** A0A2U1MWY4_ARTAN)
Solyc01g067010.3.1	-1.41	1.E-09	-1.02	3.E-05	F-box protein (AHRD V3.3 *** A0A2K3P6V3_TRIPR)
Solyc01g067300.3.1	-2.43	8.E-36	-2.36	1.E-33	CASP-like protein (AHRD V3.3 *-* A0A0K9RZ96_SPIOL)
Solyc01g080900.4.1	-2.05	5.E-22	-2.07	5.E-22	Cytochrome P450 (AHRD V3.3 *** A0A200PPF3_9MAGN)
Solyc01g088400.4.1	-1.88	0.E+00	-1.07	3.E-230	ECERIFERUM 1
Solyc01g091590.3.1	-1.68	3.E-03	-3.12	5.E-08	BON1-associated protein 2-like (AHRD V3.3 *** A0A2I4E3A5_9ROSI)
Solyc01g097110.2.1	-1.70	1.E-03	-1.81	9.E-04	Fanconi anemia group D2 protein (AHRD V3.3 *** A0A1P8B4W1_ARATH)
Solyc01g098270.1.1	-6.16	3.E-21	-7.35	1.E-22	Chaperone protein DnaJ (AHRD V3.3 *-* A0A2G2W7P0_CAPBA)
Solyc01g099150.5.1.1	-1.74	4.E-65	-1.05	8.E-26	Lipoxygenase (AHRD V3.3 *** K4B0V2_SOLLC)
Solyc01g099160.4.1	-2.04	0.E+00	-1.02	5.E-98	lipoxygenase
Solyc01g099190.4.1	-2.15	0.E+00	-1.33	0.E+00	lipoxygenase B
Solyc01g099200.3.1	-3.51	2.E-85	-4.70	2.E-119	Lipoxygenase (AHRD V3.3 *** K4B0V7_SOLLC)
Solyc01g104740.3.1	-3.25	0.E+00	-1.46	6.E-193	Multiprotein-bridging factor 1c (AHRD V3.3 *** A0A1U8F6N4_CAPAN)
Solyc01g106290.3.1	-1.08	1.E-03	-1.26	2.E-04	Unknown protein
Solyc01g107170.2.1	-1.32	9.E-21	-1.56	1.E-27	Zinc finger protein (AHRD V3.3 *** A0T3Q5_SOLTU)
Solyc01g107560.3.1	-1.72	1.E-04	-1.31	7.E-03	Glycosyltransferase (AHRD V3.3 *** A0A2G2W4I0_CAPBA)
Solyc01g107740.3.1	-1.80	2.E-35	-1.12	5.E-15	C2 domain-containing protein (AHRD V3.3 *** B1Q483_CAPCH)
Solyc01g107820.2.1	-1.32	3.E-07	-1.11	3.E-05	TOMATO WOUND-INDUCED 1
Solyc01g108250.3.1	-1.25	2.E-41	-1.00	4.E-27	DEM2
Solyc01g109160.4.1	-5.94	4.E-63	-2.73	2.E-24	CYP74C4
Solyc01g112120.4.1	-1.31	1.E-45	-1.18	2.E-37	Flavin-containing monooxygenase (AHRD V3.3 *** A0A2G2XYN9_CAPAN)
Solyc01g150144.1.1	-4.46	1.E-189	-3.40	1.E-130	Alpha/beta-Hydrolases superfamily protein (AHRD V3.3 *** A0A2U1MWY4_ARTAN)
Solyc02g065540.3.1	-1.67	3.E-19	-1.32	2.E-12	E3 ubiquitin-protein ligase (AHRD V3.3 *** A0A2G3D341_CAPCH)
Solyc02g069800.1.1	-1.64	1.E-107	-1.61	3.E-103	Alpha/beta-Hydrolases superfamily protein (AHRD V3.3 *** A0A2U1PVQ0_ARTAN)

Solyc02g073580.1.1	-3.30	1.E-14	-1.70	5.E-05	transcription factor TGA6-like (AHRD V3.3 *** A0A2I4DJ54_9ROSI)
Solyc02g077610.3.1	-2.07	3.E-44	-1.06	3.E-13	NAC domain protein (AHRD V3.3 *** S5R952_9ROSA)
Solyc02g077670.3.1	-1.85	1.E-232	-1.26	5.E-117	DnaJ-like protein (AHRD V3.3 *** Q9SP09_TOBAC)
Solyc02g081560.1.1	-1.39	2.E-04	-1.82	7.E-07	Methionyl-tRNA synthetase (AHRD V3.3 *** Q8RXY3_ARATH)
Solyc02g084410.3.1	-1.74	7.E-04	-2.89	5.E-08	Lactoylglutathione lyase (AHRD V3.3 *** A0A2K3LFU2_TRIPR)
Solyc02g084600.4.1	-1.29	1.E-05	-1.04	8.E-04	Leucine-rich repeat receptor-like protein kinase family protein (AHRD V3.3 *-* A0A061DFG6_THECC)
Solyc02g084890.3.1	-1.53	4.E-05	-2.02	8.E-08	Disease resistance RPP13-like protein 4 (AHRD V3.3 *** A0A2G2XGN6_CAPBA)
Solyc02g087280.3.1	-1.84	9.E-05	-1.71	5.E-04	Protein NRT1/ PTR FAMILY 5.9 (AHRD V3.3 *** A0A2G3D9C8_CAPCH)
Solyc02g087350.3.1	-1.08	4.E-03	-2.39	7.E-11	Hexosyltransferase (AHRD V3.3 *** A0A2G2XIE5_CAPBA)
Solyc02g088130.1.1	-2.88	1.E-18	-1.10	7.E-04	transmembrane protein (AHRD V3.3 *** AT3G29034.1)
Solyc02g089150.4.1	-1.90	7.E-28	-1.29	6.E-14	PI-PLC X domain-containing protein (AHRD V3.3 *** A0A1U8FSV1_CAPAN)
Solyc02g089160.3.1	-1.75	3.E-20	-1.77	2.E-20	dwarf
Solyc02g089610.2.1	-1.31	2.E-36	-1.07	1.E-24	S-adenosylmethionine decarboxylase 2
Solyc02g090120.1.1	-2.56	5.E-36	-1.01	2.E-07	hypothetical protein (AHRD V3.3 -** AT5G66985.1)
Solyc02g091180.1.1	-3.03	2.E-08	-2.03	2.E-04	DUF4228 domain-containing protein (AHRD V3.3 *** A0A1Q3D1C5_CEPFO)
Solyc02g091430.4.1	-1.05	2.E-15	-1.05	3.E-15	U-box domain-containing protein 4-like (AHRD V3.3 *** A0A2I4HV32_9ROSI)
Solyc02g091500.1.1	-1.58	7.E-04	-1.43	4.E-03	Calcium-dependent protein kinase (AHRD V3.3 *-* CDPK_DAUCA)
Solyc02g091700.3.1	-1.67	2.E-68	-1.57	4.E-61	Hydroxyproline-rich glycoprotein (AHRD V3.3 *** A0A2K3L507_TRIPR)
Solyc02g093420.4.1	-1.37	1.E-06	-1.13	1.E-04	NAC domain-containing protein 10 (AHRD V3.3 *** A0A2G3ACK5_CAPAN)
Solyc02g094000.1.1	-2.19	5.E-05	-3.37	3.E-09	EF_hand_5 domain-containing protein/EF_hand_6 domain-containing protein (AHRD V3.3 *** A0A1Q3B940_CEPFO)
Solyc02g094400.4.1	-1.69	7.E-68	-1.10	2.E-30	Glycerophosphodiester phosphodiesterase GDPD2 (AHRD V3.3 *** A0A2G3DA15_CAPCH)
Solyc03g005580.2.1	-6.24	1.E-08	-3.01	8.E-04	Seed storage protein (AHRD V3.3 *** Q2TPW5_9ROSI)
Solyc03g006610.3.1	-2.95	5.E-18	-1.29	1.E-04	Arf GTPase activating protein (AHRD V3.3 *** A0A200RBV9_9MAGN)
Solyc03g007430.3.1	-1.39	4.E-36	-1.12	7.E-24	Mitochondrial carrier protein MTM1 (AHRD V3.3 *** A0A2G2X7K2_CAPBA)
Solyc03g007890.3.1	-2.28	0.E+00	-2.55	0.E+00	class 2 small heat shock protein Le-HSP17.6
Solyc03g020060.3.1	-2.78	6.E-13	-1.51	8.E-05	Proteinase inhibitor type-2 (AHRD V3.3 *** A0A2G2V3L6_CAPBA)

Solyc03g025350.3.1	-3.00	9.E-13	-1.84	9.E-06	Transporter arsB (AHRD V3.3 *** A0A2U1Q5Y3_ARTAN)
Solyc03g025580.1.1	-5.28	2.E-14	-2.86	1.E-06	Pectin acetylesterase (AHRD V3.3 *** K4BF31_SOLLC)
Solyc03g025960.1.1	-1.42	9.E-04	-1.56	4.E-04	Unknown protein
Solyc03g026280.3.1	-3.03	2.E-06	-5.03	5.E-12	C-repeat binding factor 1
Solyc03g034010.3.1	-3.40	4.E-14	-4.48	7.E-19	RING-type E3 ubiquitin transferase (AHRD V3.3 *-* A0A2G3CL67_CAPCH)
Solyc03g078360.1.1	-2.24	8.E-48	-1.70	2.E-29	Receptor-like protein kinase (AHRD V3.3 *** A0A2U1PPA0_ARTAN)
Solyc03g082920.4.1	-1.40	2.E-159	-1.03	2.E-89	Heat shock protein 70 family (AHRD V3.3 *** A0A200QNK9_9MAGN)
Solyc03g083910.5.1	-4.39	0.E+00	-2.19	0.E+00	sucrose accumulator
Solyc03g093140.3.1	-1.35	5.E-121	-2.55	0.E+00	Glycerol-3-phosphate transporter 1-like protein (AHRD V3.3 *** A0A2K3PR18_TRIPR)
Solyc03g093550.1.1	-1.20	5.E-09	-3.64	7.E-57	Ethylene-responsive transcription factor 5 (AHRD V3.3 *** A0A2G3CVP7_CAPCH)
Solyc03g095700.1.1	-2.14	4.E-06	-1.94	5.E-05	hypothetical protein (AHRD V3.3 *-* AT5G05220.1)
Solyc03g096100.2.1	-4.04	2.E-11	-2.90	3.E-07	Protein yippee-like (AHRD V3.3 *** A0A1U8GAE4_CAPAN)
Solyc03g096140.2.1	-3.83	2.E-10	-3.70	1.E-09	Protein yippee-like (AHRD V3.3 *** A0A1U8GAE4_CAPAN)
Solyc03g096150.3.1	-3.86	7.E-10	-4.62	1.E-11	Protein yippee-like (AHRD V3.3 *** A0A1U8GAE4_CAPAN)
Solyc03g097440.3.1	-3.05	0.E+00	-2.23	0.E+00	11-beta-hydroxysteroid dehydrogenase 1A (AHRD V3.3 *** A0A2G3A0G7_CAPAN)
Solyc03g097670.4.1	-2.37	1.E-146	-1.90	6.E-100	DNA binding,ATP binding protein (AHRD V3.3 *** A0A2U1MGM2_ARTAN)
Solyc03g098740.1.1	-1.70	8.E-05	-1.34	3.E-03	Biotic cell death-associated protein (AHRD V3.3 *** Q850R9_NICGU)
Solyc03g112170.1.1	-3.13	2.E-11	-1.22	6.E-03	pectinesterase inhibitor-like (AHRD V3.3 *** A0A2G3A1B6_CAPAN)
Solyc03g112620.4.1	-3.55	2.E-47	-1.52	1.E-11	NEP-interacting protein, putative (DUF239) (AHRD V3.3 *** F4I5H4_ARATH)
Solyc03g113980.3.1	-2.69	5.E-05	-1.93	6.E-03	Calmodulin binding protein-like (AHRD V3.3 *** A0A200RE12_9MAGN)
Solyc03g114160.1.1	-1.91	9.E-04	-1.65	7.E-03	RING-type E3 ubiquitin transferase (AHRD V3.3 *** A0A2G2VA42_CAPBA)
Solyc03g115230.3.1	-2.49	0.E+00	-1.39	0.E+00	Solanum lycopersicum heat shock protein
Solyc03g115920.3.1	-1.61	3.E-116	-1.52	2.E-104	RBR-type E3 ubiquitin transferase (AHRD V3.3 *** A0A1U8GHK6_CAPAN)
Solyc03g116590.3.1	-1.19	3.E-37	-1.07	2.E-30	Embryo-specific protein (AHRD V3.3 *** A0A2K3LFD9_TRIPR)
Solyc03g117860.3.1	-3.00	0.E+00	-1.72	1.E-126	RBR-type E3 ubiquitin transferase (AHRD V3.3 *** A0A2G3A2R7_CAPAN)
Solyc03g117870.3.1	-1.37	4.E-92	-1.12	1.E-62	4-coumarate:CoA ligase (AHRD V3.3 *** Q42879_LITER)

Solyc03g117960.4.1	-2.10	1.E-55	-2.06	1.E-53	Forkhead-associated domain-containing protein / FHA domain-containing protein (AHRD V3.3 *** A0A2U1Q1M6_ARTAN)
Solyc03g118060.3.1	-1.35	4.E-45	-4.19	3.E-261	NFU1 iron-sulfur cluster protein (AHRD V3.3 *** AT5G07330.1)
Solyc03g118470.4.1	-2.09	2.E-10	-2.93	1.E-17	Nudix hydrolase 4 (AHRD V3.3 *** A0A2G3CZQ7_CAPCH)
Solyc03g120090.1.1	-1.94	6.E-103	-1.47	3.E-62	Pyridoxal 5'-phosphate synthase pdxS subunit (AHRD V3.3 *** U9T0K3_RHIID)
Solyc03g120900.3.1	-2.96	1.E-16	-1.15	1.E-03	WD40 repeat (AHRD V3.3 *** A0A200QDT5_9MAGN)
Solyc03g120990.3.1	-1.61	2.E-07	-1.41	9.E-06	Malic enzyme (AHRD V3.3 *** A0A2G3D1G2_CAPCH)
Solyc03g121000.3.1	-1.15	3.E-09	-1.13	1.E-08	Protein PAF1-like protein (AHRD V3.3 *-* A0A2G3A3W1_CAPAN)
Solyc03g121890.1.1	-2.10	1.E-131	-1.15	3.E-44	Hydroxyproline-rich glycoprotein family protein (AHRD V3.3 *-* Q9CAJ1_ARATH)
Solyc03g123540.3.1	-1.80	6.E-77	-3.04	1.E-173	17.4 kDa class III heat shock protein (AHRD V3.3 *** A0A2G3D1F4_CAPCH)
Solyc03g123620.4.1	-1.66	3.E-04	-1.66	4.E-04	Pectinesterase (AHRD V3.3 *** A0A2G3D1E1_CAPCH)
Solyc04g005480.1.1	-1.00	3.E-03	-1.35	5.E-05	nodulin-related protein 1-like (AHRD V3.3 *** A0A2I4FXG1_9ROSI)
Solyc04g009900.4.1	-1.09	2.E-04	-1.77	2.E-09	phosphoenolpyruvate carboxylase kinase
Solyc04g009910.3.1	-1.18	3.E-03	-1.55	1.E-04	Phosphoenolpyruvate carboxylase kinase (AHRD V3.3 *** Q9SDY0_SOLLC)
Solyc04g051490.3.1	-1.85	2.E-173	-1.74	5.E-156	Crossover junction endonuclease EME1B (AHRD V3.3 *** A0A2G2VFP4_CAPBA)
Solyc04g064850.3.1	-1.07	4.E-07	-1.05	1.E-06	Protein LAZ1-like protein 1 (AHRD V3.3 *** A0A2G2WZ08_CAPBA)
Solyc04g071030.1.1	-1.56	2.E-08	-1.77	3.E-10	RING-type E3 ubiquitin transferase (AHRD V3.3 *** A0A2G2WYL4_CAPBA)
Solyc04g071480.1.1	-1.84	8.E-19	-1.89	2.E-19	Plant/F1M20-13 protein (AHRD V3.3 *** G7IBZ7_MEDTR)
Solyc04g077850.3.1	-1.83	4.E-10	-1.06	6.E-04	RING-type E3 ubiquitin transferase (AHRD V3.3 *-* A0A2U1KPH2_ARTAN)
Solyc04g077980.1.1	-1.58	4.E-10	-2.98	3.E-29	C2H2-type zinc finger protein
Solyc04g079190.4.1	-1.19	3.E-05	-1.34	4.E-06	Alpha/beta-Hydrolases superfamily protein (AHRD V3.3 *** A0A2U1KK17_ARTAN)
Solyc04g082960.1.1	-1.54	2.E-07	-2.91	3.E-21	Poly polymerase (AHRD V3.3 *** Q9C9J8_ARATH)
Solyc05g005260.3.1	-1.20	2.E-05	-1.53	9.E-08	DNA ligase 1-like (AHRD V3.3 *** A0A1U7VHC8_NICSY)
Solyc05g005290.4.1	-2.31	2.E-134	-1.83	1.E-89	Poly [ADP-ribose] polymerase (AHRD V3.3 *** A0A2G2WJW6_CAPBA)
Solyc05g005460.3.1	-2.09	8.E-53	-2.04	4.E-50	DC1 domain-containing protein (AHRD V3.3 *** A0A2U1QM98_ARTAN)
Solyc05g005910.4.1	-5.10	0.E+00	-4.31	0.E+00	Protein NRT1/ PTR FAMILY 1.2 (AHRD V3.3 *-* A0A1J3FWJ0_NOCCA)
Solyc05g010420.2.1	-1.48	0.E+00	-1.05	0.E+00	S-adenosylmethionine decarboxylase

Solyc05g023995.1.1	-1.41	3.E-66	-1.19	2.E-48	Unknown protein
Solyc05g046310.3.1	-1.21	6.E-35	-1.12	6.E-30	DBH-like monooxygenase (AHRD V3.3 *** AT5G35320.1)
Solyc05g051220.3.1	-1.02	3.E-09	-1.45	9.E-17	Potassium channel GORK (AHRD V3.3 *** A0A1U8GYE0_CAPAN)
Solyc05g052040.1.1	-1.04	3.E-11	-2.28	1.E-45	ripening regulated protein DDTFR10/A
Solyc05g052550.1.1	-1.46	2.E-12	-2.79	2.E-37	Zinc finger CCCH domain-containing protein 47 (AHRD V3.3 *-* A0A1U8GQ71_CAPAN)
Solyc05g052570.3.1	-1.31	1.E-25	-2.58	3.E-85	Zinc finger transcription factor 39
Solyc05g052950.4.1	-2.90	3.E-30	-1.80	2.E-14	Regulator of chromosome condensation (RCC1) family protein (AHRD V3.3 *** Q9M2S1_ARATH)
Solyc05g053290.3.1	-1.78	1.E-22	-1.06	6.E-09	Protein phosphatase-2C (AHRD V3.3 *** O82469_MESCR)
Solyc05g053500.4.1	-1.09	1.E-03	-1.44	1.E-05	GATA transcription factor (AHRD V3.3 *** G7K6G0_MEDTR)
Solyc05g053620.3.1	-2.28	6.E-50	-1.65	3.E-28	F-box protein PP2-B11-like (AHRD V3.3 *** Q3Y625_TOBAC)
Solyc05g055010.4.1	-1.35	8.E-44	-1.22	1.E-35	RNA-binding protein 42 (AHRD V3.3 *** A0A2I4E3H6_9ROSI)
Solyc05g055870.3.1	-1.52	3.E-24	-1.17	5.E-15	F-box domain, Phloem protein 2-like protein (AHRD V3.3 *** A0A2U1PQ05_ARTAN)
Solyc05g056390.2.1	-1.45	4.E-89	-1.08	5.E-51	10 kDa chaperonin (AHRD V3.3 *** A0A1U8F6X5_CAPAN)
Solyc06g005650.2.1	-1.68	7.E-20	-1.37	1.E-13	E3 ubiquitin-protein ligase (AHRD V3.3 *** A0A1U8GX66_CAPAN)
Solyc06g007190.4.1	-2.02	5.E-03	-2.29	2.E-03	Protein phosphatase 2C (AHRD V3.3 *** O24078_MEDSA)
Solyc06g008870.2.1	-1.54	3.E-47	-1.32	2.E-35	Gibberellin receptor (AHRD V3.3 *** E3VJP7_9FABA)
Solyc06g035940.3.1	-1.55	2.E-70	-4.04	0.E+00	Homeobox-leucine zipper protein ROC5 (AHRD V3.3 *** A0A2G2X6G6_CAPBA)
Solyc06g035960.3.1	-1.09	6.E-04	-1.38	2.E-05	Oxalate--CoA ligase (AHRD V3.3 *** A0A1U8FIW0_CAPAN)
Solyc06g036290.3.1	-3.46	0.E+00	-2.49	0.E+00	heat shock protein 90
Solyc06g053620.4.1	-1.34	1.E-62	-1.44	6.E-71	phosphoenolpyruvate carboxylase kinase 2
Solyc06g062540.3.1	-1.09	9.E-06	-2.37	3.E-21	phosphatase (psi14C gene)
Solyc06g065010.4.1	-2.60	1.E-09	-1.85	2.E-05	GDSL esterase/lipase 2-like (AHRD V3.3 *** A0A2G2ZB04_CAPAN)
Solyc06g071580.3.1	-2.78	2.E-26	-3.33	9.E-34	ATPase, histidine kinase, DNA gyrase B-, and HSP90-like domain containing protein, expressed (AHRD V3.3 *** Q33AG5_ORYSJ)
Solyc06g072330.3.1	-1.03	3.E-03	-1.77	2.E-07	Mitochondrial import inner membrane translocase subunit TIM14 (AHRD V3.3 *** B6T323_MAIZE)

Solyc06g072430.3.1	-2.38	2.E-277	-1.00	5.E-59	BAG family molecular chaperone regulator 5, mitochondrial (AHRD V3.3 *-* A0A2I0XHJ2_9ASPA)
Solyc06g073030.3.1	-3.10	2.E-14	-1.12	7.E-03	Stomatin family protein (SPFH domain/Band 7 family protein) (AHRD V3.3 *** A0A166USA6_9PEZI)
Solyc06g074030.1.1	-1.16	9.E-06	-2.68	7.E-24	Polynucleotidyl transferase, ribonuclease H-like superfamily protein (AHRD V3.3 *** A0A2U1M9A7_ARTAN)
Solyc06g075775.1.1	-3.97	2.E-13	-2.20	1.E-05	Zinc finger protein ZAT12 (AHRD V3.3 *** A0A2G3CB25_CAPCH)
Solyc06g075780.3.1	-3.00	6.E-55	-2.09	2.E-30	Zinc finger protein (AHRD V3.3 *** A0A200PZ96_9MAGN)
Solyc06g076020.3.1	-4.34	0.E+00	-2.31	2.E-294	heat shock protein 70 kD
Solyc06g076540.1.1	-2.03	0.E+00	-1.97	0.E+00	Class I heat shock protein (AHRD V3.3 *** A0A2K3M3P7_TRIPR)
Solyc06g076670.3.1	-1.51	3.E-221	-1.27	4.E-161	RNA-binding (RRM/RBD/RNP motifs) family protein (AHRD V3.3 *-* A0A1P8AVT0_ARATH)
Solyc06g083190.4.1	-1.82	0.E+00	-1.32	0.E+00	Peptidylprolyl isomerase (AHRD V3.3 *** A0A2G3CCB5_CAPCH)
Solyc06g150138.1.1	-2.80	5.E-77	-1.78	1.E-34	Unknown protein
Solyc07g006050.3.1	-1.26	2.E-04	-1.23	5.E-04	Histone-lysine N-methyltransferase ASHR3 (AHRD V3.3 *-* A0A2G2XZF7_CAPAN)
Solyc07g007590.1.1	-1.36	1.E-04	-1.86	2.E-07	arogenate dehydrogenase 2, chloroplastic (AHRD V3.3 *** A0A2G2Z0J0_CAPAN)
Solyc07g008620.1.1	-1.80	6.E-24	-1.97	1.E-27	EIX receptor 1
Solyc07g017780.4.1	-1.23	7.E-32	-1.08	1.E-24	(DB226) meloidogyne-induced giant cell protein
Solyc07g040960.1.1	-1.89	2.E-45	-3.56	7.E-129	Harbinger transposase-derived nuclease (AHRD V3.3 *** A0A200QD47_9MAGN)
Solyc07g042400.2.1	-1.81	3.E-05	-1.95	1.E-05	Unknown protein
Solyc07g042480.3.1	-5.08	1.E-56	-5.22	2.E-56	Unknown protein
Solyc07g043590.3.1	-1.14	6.E-62	-1.46	2.E-97	Polyamine oxidase 3 (AHRD V3.3 *** A0A2G3BYY2_CAPCH)
Solyc07g044990.3.1	-1.04	7.E-16	-1.08	9.E-17	TRAM, LAG1 and CLN8 (TLC) lipid-sensing domain containing protein (AHRD V3.3 *** A0A2U1Q6S5_ARTAN)
Solyc07g049530.3.1	-3.10	0.E+00	-1.22	0.E+00	1-aminocyclopropane-1-carboxylate oxidase 1
Solyc07g053740.1.1	-1.00	2.E-08	-1.46	4.E-16	Ethylene Response Factor F.4
Solyc07g054810.1.1	-2.14	2.E-05	-1.65	2.E-03	Unknown protein
Solyc07g056000.2.1	-1.00	3.E-03	-1.75	9.E-08	Xyloglucan endotransglucosylase/hydrolase (AHRD V3.3 *** Q43528_SOLLC)
Solyc07g061800.4.1	-1.73	1.E-10	-3.85	2.E-35	Heme-binding-like protein (AHRD V3.3 *** A0A2I0X1C8_9ASPA)

Solyc07g064410.1.1	-2.20	6.E-09	-3.15	9.E-14	Fatty acid desaturase 4, chloroplastic (AHRD V3.3 *** A0A1U8FLY9_CAPAN)
Solyc07g066370.3.1	-1.36	3.E-08	-1.26	5.E-07	SNARE-associated protein-like protein (AHRD V3.3 *** AT1G79070.2)
Solyc08g005050.4.1	-1.08	3.E-09	-1.81	4.E-23	transcription factor MYC2 (AHRD V3.3 *-* R4S7B4_NICAT)
Solyc08g008370.3.1	-1.12	2.E-34	-1.48	4.E-57	DCD (Development and Cell Death) domain protein (AHRD V3.3 *-* A0A2U1P2J5_ARTAN)
Solyc08g041860.1.1	-1.08	8.E-18	-1.41	5.E-29	serine/arginine repetitive matrix-like protein (AHRD V3.3 *-* AT4G32020.1)
Solyc08g060920.4.1	-1.22	1.E-49	-2.01	2.E-122	IDS4-like
Solyc08g061590.4.1	-1.30	9.E-20	-1.27	2.E-18	Pentatricopeptide repeat-containing protein (AHRD V3.3 *** A0A2G2VV98_CAPBA)
Solyc08g062220.3.1	-1.39	2.E-13	-1.51	4.E-15	Glycosyltransferase (AHRD V3.3 *** A0A218W5V3_PUNGR)
Solyc08g062680.1.1	-1.96	9.E-05	-1.58	3.E-03	Harbinger transposase-derived nuclease (AHRD V3.3 *-* A0A2U1Q453_ARTAN)
Solyc08g068070.3.1	-1.17	2.E-15	-1.07	9.E-13	Two-on-two hemoglobin-3 (AHRD V3.3 *** A0A2G3BSD0_CAPCH)
Solyc08g075540.5.1.1	-2.45	8.E-286	-1.53	8.E-126	alternative oxidase
Solyc08g076390.3.1	-1.32	3.E-03	-2.77	1.E-09	Transcription factor jumonji (Jmj) family protein / zinc finger (C5HC2 type) family protein (AHRD V3.3 *** F4KIX0_ARATH)
Solyc08g077980.4.1	-1.75	1.E-204	-1.51	6.E-156	Bax inhibitor
Solyc08g078190.2.1	-1.50	6.E-06	-3.22	5.E-20	Ethylene-responsive transcription factor 5 (AHRD V3.3 *** A0A2G2XL45_CAPBA)
Solyc08g078700.2.1	-2.67	0.E+00	-1.69	0.E+00	mitochondrial small heat shock protein
Solyc08g079170.3.1	-1.31	6.E-253	-1.21	4.E-218	Hsp70-Hsp90 organizing protein 1 (AHRD V3.3 *** A0A1U8GC69_CAPAN)
Solyc08g079700.3.1	-2.39	7.E-149	-1.18	9.E-43	Zinc finger A20 and AN1 domain stress-associated protein (AHRD V3.3 *** A0A2K3LX56_TRIPR)
Solyc08g080540.3.1	-1.63	9.E-29	-1.33	1.E-19	Heat stress transcription factor B-2b (AHRD V3.3 *** A0A2G3DB98_CAPCH)
Solyc08g080650.3.1	-1.49	0.E+00	-1.76	0.E+00	PATHOGENESIS RELATED PROTEIN P23
Solyc08g081010.3.1	-1.34	3.E-157	-1.05	6.E-99	gamma-glutamylcysteine synthetase 1
Solyc08g082090.1.1	-1.21	6.E-07	-2.24	1.E-19	Avr9/Cf-9 rapidly elicited protein 194 (AHRD V3.3 *** Q9FQZ4_TOBAC)
Solyc08g083050.1.1	-3.37	7.E-67	-1.64	2.E-20	Erythronate-4-phosphate dehydrogenase family protein (AHRD V3.3 -** AT1G19400.3)
Solyc09g005120.3.1	-2.25	7.E-138	-1.32	8.E-54	DnaJ protein ERDJ3A (AHRD V3.3 *** A0A2G2VZ69_CAPBA)
Solyc09g005260.4.1	-2.14	5.E-32	-2.15	5.E-32	Vacuolar cation/proton exchanger (AHRD V3.3 *** A0A2G3BK94_CAPCH)
Solyc09g006005.1.1	-5.49	6.E-14	-2.96	3.E-06	Pathogenesis-related protein 1 (AHRD V3.3 *** Q75QH2_CAPCH)

Solyc09g007010.1.1	-6.44	3.E-13	-2.46	4.E-04	Pathogenesis-related protein 1 (AHRD V3.3 *** Q75QH2_CAPCH)
Solyc09g007860.4.1	-1.23	1.E-16	-1.23	3.E-16	Calcium-dependent lipid-binding (CaLB domain) family protein (AHRD V3.3 *** A0A1P8BD17_ARATH)
Solyc09g008970.1.1	-1.48	1.E-160	-2.41	0.E+00	plant/protein (AHRD V3.3 *** AT3G10020.1)
Solyc09g015770.3.1	-1.12	2.E-08	-1.91	8.E-21	WRKY transcription factor 81
Solyc09g019970.3.1	-1.06	3.E-24	-1.09	2.E-25	Ubiquitin carboxyl-terminal hydrolase (AHRD V3.3 *** S8E5A0_9LAMI)
Solyc09g061310.3.1	-1.56	1.E-125	-1.15	2.E-71	PPPDE putative thiol peptidase family protein (AHRD V3.3 *** A0A2U1N5E2_ARTAN)
Solyc09g063010.4.1	-1.53	6.E-15	-1.10	4.E-08	bHLH transcription factor 058
Solyc09g064380.3.1	-1.37	2.E-34	-1.05	8.E-21	Tetratricopeptide repeat (TPR)-like superfamily protein (AHRD V3.3 *** F4I456_ARATH)
Solyc09g064940.2.1	-2.83	0.E+00	-1.79	0.E+00	Phenazine biosynthesis PhzF protein (AHRD V3.3 *** A0A200Q4V2_9MAGN)
Solyc09g065610.2.1	-1.64	9.E-04	-1.69	9.E-04	Bax inhibitor-1 family protein (AHRD V3.3 *-* A0A2U1MW73_ARTAN)
Solyc09g075140.3.1	-1.71	2.E-06	-1.16	2.E-03	Alpha/beta-Hydrolases superfamily protein (AHRD V3.3 *** A0A2U1LF81_ARTAN)
Solyc09g075950.1.1	-1.37	9.E-121	-1.94	8.E-223	Heat shock protein 70 kDa (AHRD V3.3 *** A0A2K3P5L8_TRIPR)
Solyc09g082230.1.1	-2.32	3.E-11	-2.15	1.E-09	N-acetyltransferase (AHRD V3.3 *** A0A2N9Z4V7_PAEPO)
Solyc09g082240.4.1	-2.24	2.E-15	-1.36	2.E-06	N-acetyltransferase (AHRD V3.3 *-* A0A2N9Z4V7_PAEPO)
Solyc09g082250.3.1	-2.84	2.E-41	-2.77	5.E-39	N-acetyltransferase (AHRD V3.3 *** A0A2N9Z4V7_PAEPO)
Solyc09g082690.3.1	-1.15	1.E-69	-2.17	8.E-231	superoxide dismutase
Solyc09g082720.3.1	-1.22	2.E-37	-1.51	5.E-55	NAD(P)-linked oxidoreductase, aldo/keto reductase family protein (AHRD V3.3 *** A0A1Y1HY98_KLENI)
Solyc09g082810.3.1	-3.12	6.E-52	-2.72	2.E-41	Unknown protein
Solyc09g091180.3.1	-1.13	5.E-74	-1.25	5.E-88	60 kDa chaperonin (AHRD V3.3 *** Q8H903_ORYSJ)
Solyc09g092480.1.1	-1.29	2.E-07	-1.40	2.E-08	Glycosyltransferase (AHRD V3.3 *** A0A2I4GJH4_9ROSI)
Solyc09g092763.1.1	-5.38	9.E-37	-2.50	1.E-11	DNA-directed RNA polymerase subunit beta'' (AHRD V3.3 *-* A0A2H4NAQ9_9ORYZ)
Solyc09g092765.1.1	-5.12	1.E-132	-2.35	5.E-45	DNA-directed RNA polymerase subunit beta'' (AHRD V3.3 *-* A0A2H4NAQ9_9ORYZ)
Solyc09g092767.1.1	-5.16	5.E-34	-2.34	8.E-10	DNA-directed RNA polymerase subunit beta'' (AHRD V3.3 *-* A0A2H4NAQ9_9ORYZ)

Solyc09g098050.2.1	-2.68	1.E-10	-1.84	8.E-06	Ankyrin repeat (AHRD V3.3 *** A0A200PSP7_9MAGN)
Solyc10g006130.1.1	-1.33	1.E-11	-1.94	2.E-22	EAR motif SlERF36
Solyc10g006660.3.1	-1.16	3.E-08	-2.30	4.E-27	Calcium-binding protein PBP1 (AHRD V3.3 *** A0A1U8EDM6_CAPAN)
Solyc10g006800.4.1	-1.93	2.E-04	-2.42	4.E-06	Cyclic nucleotide-gated ion channel 4 (AHRD V3.3 *** A0A2G2V344_CAPBA)
Solyc10g008400.1.1	-1.84	4.E-26	-1.38	2.E-15	E3 ubiquitin-protein ligase RMA1H1 (AHRD V3.3 *** A0A2G3BCU0_CAPCH)
Solyc10g009110.1.1	-1.54	5.E-15	-1.59	1.E-15	ethylene response factor F.5
Solyc10g047030.3.1	-4.63	0.E+00	-3.10	1.E-187	LEXYL1
Solyc10g047040.3.1	-2.02	1.E-11	-1.41	3.E-06	Zinc finger transcription factor 58
Solyc10g050990.2.1	-3.01	5.E-49	-1.49	3.E-15	Unknown protein
Solyc10g051020.2.1	-1.71	8.E-71	-1.49	2.E-54	Cytochrome P450 (AHRD V3.3 *** A0A2U1MAN7_ARTAN)
Solyc10g075090.3.1	-3.45	0.E+00	-1.36	2.E-186	Non-specific lipid-transfer protein (AHRD V3.3 *** K4D1V1_SOLLC)
Solyc10g075103.1.1	-3.45	0.E+00	-1.35	2.E-185	Non-specific lipid-transfer protein (AHRD V3.3 *** K4D1V1_SOLLC)
Solyc10g076380.2.1	-4.81	1.E-15	-1.77	6.E-04	Dehydration responsive element binding transcription factor (AHRD V3.3 *-* W6FJ00_9ROSA)
Solyc10g078930.2.1	-1.66	1.E-216	-1.65	6.E-214	Activator of 90 kDa heat shock protein ATPase-like protein 1 (AHRD V3.3 *** A0A1J3GDI7_NOCCA)
Solyc10g080210.2.1	-3.44	0.E+00	-2.33	0.E+00	polygalacturonase
Solyc10g081930.1.1	-2.83	2.E-26	-1.16	7.E-06	Cytoplasmic FMR1 interacting protein (AHRD V3.3 *-* A0A1Y1I7X8_KLENI)
Solyc10g081940.2.1	-2.71	2.E-20	-1.12	1.E-04	Exocyst subunit Exo70 family protein (AHRD V3.3 *** A0A1S3WZ57_TOBAC)
Solyc10g081980.2.1	-1.63	5.E-10	-1.06	1.E-04	Late embryogenesis abundant (LEA) hydroxyproline-rich glycoprotein family (AHRD V3.3 *** A0A2U1KP17_ARTAN)
Solyc10g084170.1.1	-3.06	1.E-64	-1.50	3.E-20	Bag family molecular chaperone regulator mitochondrial-like (AHRD V3.3 *** A0A2K3P7D1_TRIPR)
Solyc10g084880.3.1	-1.76	2.E-07	-2.40	4.E-12	Avr9/Cf-9 rapidly elicited protein 137 (AHRD V3.3 *** Q9FQZ2_TOBAC)
Solyc10g085040.1.1	-1.34	9.E-107	-1.18	1.E-82	Heme-binding-like protein (AHRD V3.3 *** A0A2I0X8X3_9ASPA)
Solyc10g085850.1.1	-1.77	2.E-12	-1.66	7.E-11	TPSI1 protein (AHRD V3.3 *** Q41336_SOLLC)
Solyc10g086410.3.1	-2.55	2.E-247	-1.88	2.E-147	LEHSC270 hsc-2heat shock protein cognate 70
Solyc11g005740.2.1	-2.79	3.E-04	-2.18	7.E-03	RING/FYVE/PHD zinc finger superfamily protein (AHRD V3.3 *-* B3H578_ARATH)
Solyc11g005750.2.1	-2.95	4.E-08	-2.82	3.E-07	Pectinesterase (AHRD V3.3 *** A0A2I4GT13_9ROSI)

Solyc11g006740.3.1	-2.59	2.E-41	-1.74	6.E-21	F-box domain, Phloem protein 2-like protein (AHRD V3.3 *** A0A2U1NHQ2_ARTAN)
Solyc11g008530.3.1	-2.82	0.E+00	-1.38	1.E-212	Dicer-like 2d
Solyc11g008540.3.1	-3.03	0.E+00	-1.27	8.E-151	Dicer-like 2b
Solyc11g044470.3.1	-1.22	2.E-04	-1.05	2.E-03	RNA-binding (RRM/RBD/RNP motifs) family protein (AHRD V3.3 *** Q67YS0_ARATH)
Solyc11g066100.2.1	-2.13	0.E+00	-1.75	0.E+00	Heat shock protein 70 (AHRD V3.3 *** S8C6K2_9LAMI)
Solyc11g066400.1.1	-3.79	2.E-08	-2.71	4.E-05	Zinc finger protein (AHRD V3.3 *** A0A200R5F9_9MAGN)
Solyc11g069700.2.1	-2.56	0.E+00	-1.52	2.E-253	Elongation factor 1-alpha (AHRD V3.3 *** A0A1U8EL12_CAPAN)
Solyc11g071830.2.1	-2.60	0.E+00	-2.44	2.E-277	DnaJ protein like (AHRD V3.3 *** A0A2I0X6P9_9ASPA)
Solyc12g009000.1.1	-1.98	4.E-23	-3.31	1.E-53	Harbinger transposase-derived nuclease (AHRD V3.3 *** A0A2U1Q453_ARTAN)
Solyc12g009480.2.1	-1.48	1.E-14	-1.99	3.E-24	SPX domain-containing protein (AHRD V3.3 *** A0A1Q3BK26_CEPFO)
Solyc12g015880.2.1	-2.37	0.E+00	-1.14	7.E-188	Heat shock protein 90-1
Solyc12g062200.1.1	-1.72	3.E-12	-2.10	7.E-17	Unknown protein
Solyc12g089210.2.1	-1.32	2.E-13	-1.42	4.E-15	Ornithine carbamoyltransferase (AHRD V3.3 *** A0A2G2YA09_CAPAN)
Solyc12g098710.2.1	-1.38	2.E-154	-1.10	2.E-100	15-cis-zeta-carotene isomerase, chloroplastic (AHRD V3.3 *** A0A1U8ESN0_CAPAN)
